# Relaxin-3/RXFP3 networks: an emerging target for the treatment of depression and other neuropsychiatric diseases?

**DOI:** 10.3389/fphar.2014.00046

**Published:** 2014-03-21

**Authors:** Craig M. Smith, Andrew W. Walker, Ihaia T. Hosken, Berenice E. Chua, Cary Zhang, Mouna Haidar, Andrew L. Gundlach

**Affiliations:** ^1^Peptide Neurobiology Laboratory, Neuropeptides Division, The Florey Institute of Neuroscience and Mental Health, The University of MelbourneVIC, Australia; ^2^Florey Department of Neuroscience and Mental Health, The University of MelbourneVIC, Australia; ^3^Department of Anatomy and Neuroscience, The University of MelbourneVIC, Australia

**Keywords:** relaxin-3, RXFP3, neuropeptide, arousal, stress, mood and depression, autism spectrum disorders, eating disorders

## Abstract

Animal and clinical studies of gene-environment interactions have helped elucidate the mechanisms involved in the pathophysiology of several mental illnesses including anxiety, depression, and schizophrenia; and have led to the discovery of improved treatments. The study of neuropeptides and their receptors is a parallel frontier of neuropsychopharmacology research and has revealed the involvement of several peptide systems in mental illnesses and identified novel targets for their treatment. Relaxin-3 is a newly discovered neuropeptide that binds, and activates the G-protein coupled receptor, RXFP3. Existing anatomical and functional evidence suggests relaxin-3 is an arousal transmitter which is highly responsive to environmental stimuli, particularly neurogenic stressors, and in turn modulates behavioral responses to these stressors and alters key neural processes, including hippocampal theta rhythm and associated learning and memory. Here, we review published experimental data on relaxin-3/RXFP3 systems in rodents, and attempt to highlight aspects that are relevant and/or potentially translatable to the etiology and treatment of major depression and anxiety. Evidence pertinent to autism spectrum and metabolism/eating disorders, or related psychiatric conditions, is also discussed. We also nominate some key experimental studies required to better establish the therapeutic potential of this intriguing neuromodulatory signaling system, including an examination of the impact of RXFP3 agonists and antagonists on the overall activity of distinct or common neural substrates and circuitry that are identified as dysfunctional in these debilitating brain diseases.

## INTRODUCTION

It has now become widely accepted by neuroscientists and the clinical community that mental illness can arise from multiple sources and causes, including genetic mutations or epigenetic effects, and key environmental impacts during early development, and adolescence. A need for an ongoing reappraisal of how best to study and classify mental illness is also acknowledged, including the development of circuit-level frameworks for understanding different modality deficits in depression (e.g., Nestler, 1998; Willner et al., 2013), autism spectrum disorders (ASD; e.g., Haznedar et al., 2000; Markram and Markram, 2010; Yizhar et al., 2011b; Fan et al., 2012), and schizophrenia (e.g., Spencer et al., 2003; O’Donnell, 2011; Millan et al., 2012; Jiang et al., 2013).

Similarly, novel structural and molecular targets in brain that might underpin better treatments for the debilitating conditions encompassed by the clinical spectrum of anxiety, major depression, and related psychiatric illnesses need to be identified and explored. In this regard, it is clear that neuromodulatory systems that utilize monoamine and peptide transmitters play a key role in the neurophysiology of circuits associated with affective behavior and cognition (Hoyer and Bartfai, 2012; Marder, 2012; van den Pol, 2012), and they can be both aberrant in psychiatric pathology and targets for novel treatments (e.g., Domschke et al., 2011; Hoyer and Bartfai, 2012; Lin and Sibille, 2013).

Relaxin-3 is a highly conserved neuropeptide that is abundantly expressed in four small groups of largely γ-aminobutyric acid (GABA) projection neurons in mammalian brain (Bathgate et al., 2002; Burazin et al., 2002; Tanaka et al., 2005), and is involved in regulating aspects of physiological and behavioral stress responses and the integration of sensory inputs (see Smith et al., 2011). Recent reviews have highlighted the putative role of relaxin-3 in the control of feeding and the neuroendocrine axis (Tanaka, 2010; Ganella et al., 2012, 2013b). However, existing neuroanatomical and functional evidence also suggests the GABA/relaxin-3 system acts as a broad “arousal” network which is highly responsive to environmental stimuli (neurogenic stressors) and modulates stress responses and other key behaviors/neural processes. These effects are mediated via a variety of mechanisms, such as influencing hippocampal theta rhythm and associated learning and memory, and via putative actions throughout the limbic system (Tanaka et al., 2005; Ma et al., 2009a, 2013; Banerjee et al., 2010). Here, in the broader context of the potential for neuropeptide-receptor systems as therapeutic drug targets (Hoyer and Bartfai, 2012), we review existing experimental data on relaxin-3 and modulation of its receptor, relaxin family peptide 3 receptor (RXFP3), in rodents and highlight its relevance to the etiology of various neuropsychiatric disorders.

## NEUROPEPTIDE-RECEPTOR SYSTEMS AS TARGETS FOR TREATMENT OF NEUROPSYCHIATRIC DISORDERS

Since the early discovery of “substance P” (von Euler and Gaddum, 1931), a plethora of neuropeptide-receptor systems have been identified and characterized (see Hoyer and Bartfai, 2012). Neuropeptides are commonly co-released with GABA/glutamate and monoamine transmitters, and generally signal through G-protein coupled receptors to modulate a broad range of neural processes and behaviors. The potential attractiveness of neuropeptide-receptor systems as therapeutic drug targets is enhanced by their high level of signaling specificity. For example, expression of neuropeptides is often restricted to small populations of neurons within a small number of brain nuclei (e.g., orexin, MCH, and neuropeptide S; Xu et al., 2004; Sakurai, 2007; Saito and Nagasaki, 2008), and neuropeptides frequently bind to their receptors with high affinity and specificity due to their generally large allosteric binding sites (Hoyer and Bartfai, 2012). Neuropeptides are also often preferentially released under states of high neuronal firing frequency in response to the nervous system being challenged, as can occur during acute or chronic environmental stress and/or in association with neuropsychiatric disorders (Hökfelt et al., 2000, 2003; Holmes et al., 2003).

These characteristics suggest that therapeutic drugs which target neuropeptide systems may be less prone to unwanted “non-specific” side-effects compared to current drug treatments. For example, although tricyclic antidepressants are relatively effective at increasing 5-hydroxytrypamine (5-HT) and noradrenaline signaling to reduce the symptoms of major depression, they are hampered by cross-reactivity with other transmitter systems and reduce histamine and cholinergic signaling, which contributes to unwanted side effects (Westenberg, 1999). Even their “replacement” drugs (selective serotonin reuptake inhibitors, SSRIs) are associated with shortcomings such as slow onset of action and patient resistance, and side effects including sexual dysfunction, and weight gain (Nestler, 1998). Similar problems have been encountered in the development of antipsychotics to treat schizophrenia (Tandon, 2011), suggesting that more selective drugs that target relevant peptide receptors could have broad therapeutic applications (Hökfelt et al., 2003; Holmes et al., 2003; Hoyer and Bartfai, 2012).

Interest in the therapeutic potential of neuropeptide-receptor systems has further increased following a number of studies which implicate their dysregulation as contributing to disease susceptibility. For example, narcolepsy is strongly associated with reduced orexin signaling (Burgess and Scammell, 2012); post-traumatic stress syndrome (PTSD) susceptibility and panic has been linked to pituitary adenylate cyclase-activating polypeptide (PACAP) receptor-1 and corticotrophin-releasing factor (CRF) receptor-2 signaling (Ressler et al., 2011; Lebow et al., 2012; see also Dore et al., 2013); and neuropeptide Y (NPY) and CRF appear to play a role not only in the underlying pathophysiology of schizophrenia and depression, but as likely downstream mediators of the therapeutic effects following treatment with monoamine-targeting drugs (Arborelius et al., 1999; Ishida et al., 2007; Zorrilla and Koob, 2010; Nikisch et al., 2011). Not surprisingly, the antidepressant potential of drugs which directly target NPY and CRF signaling is currently under investigation (Paez-Pereda et al., 2011), while drugs that target receptors for neurotrophic factors and other neuropeptides, such as brain-derived neurotrophic factor (BDNF; Vithlani et al., 2013) and neuropeptide S (NPS; Pape et al., 2010), offer considerable promise as antidepressants and anxiolytics (Schmidt and Duman, 2010; McGonigle, 2011), in light of the effects of the native peptides in relevant animal models of neurogenesis, and neural structure and activity (Rotzinger et al., 2010; Pulga et al., 2012).

However, from a translational viewpoint, over the last two decades pharmaceutical and biotechnology groups have been attempting to target neuropeptide systems to treat various CNS disorders and despite encouraging pre-clinical data, clinical studies investigating the antidepressant potential of neuropeptide receptor-targeting drugs have yielded mixed findings. For example, the neurokinin 1 (NK_1_) antagonist “aprepitant,” which is effective at treating nausea during chemotherapy (de Wit et al., 2004), was unsuccessful in the treatment of major depression (Keller et al., 2006). CRF receptor-1 antagonists are also yet to demonstrate clear antidepressant properties (Binneman et al., 2008), although anxiolytic effects are promising (Bailey et al., 2011); and trials of these compounds against alcohol abuse and relapse are being undertaken (Zorrilla et al., 2013). NPY agonists were initially observed to inhibit circulating stress hormones during sleep in healthy controls (Antonijevic et al., 2000), while subsequent testing in depressed patients failed to confer therapeutic effects (Held et al., 2006). Although frustrating for industry *and* clinical and basic researchers, in regard to depression, these findings are more likely to reflect the complex underlying nature of the targeted disorder and its symptoms, rather than inherent flaws with neuropeptide-receptor systems as drug targets. Indeed, more recently, drugs that target orexin receptors have demonstrated promise in the treatment of sleep disorders (Hoyer and Jacobson, 2013; Winrow and Renger, 2014).

## THE NEUROPEPTIDE RELAXIN-3 AND ITS RECEPTOR, RXFP3

Relaxin-3 is a two chain, 51 amino acid neuropeptide discovered by our laboratory in 2001 (Bathgate et al., 2002; Burazin et al., 2002; Rosengren et al., 2006). Relaxin-3 is the ancestral gene of the relaxin family of peptides (Wilkinson et al., 2005), which includes the namesake peptide “relaxin” (H2 relaxin or relaxin-2 in humans) that was observed to relax the pelvic ligament in guinea pigs almost a century ago (Hisaw, 1926). In contrast to the many and varied peripheral actions of relaxin (Sherwood, 2004; Bathgate et al., 2013a), relaxin-3 is abundantly expressed within the mammalian brain (Bathgate et al., 2002; Burazin et al., 2002) and acts as a neurotransmitter by activating its cognate G-protein coupled receptor, RXFP3 [also known as GPCR135, SALPR, and GPR100; Matsumoto et al., 2000; Liu et al., 2003; Boels et al., 2004; see Bathgate et al., 2006, 2013a]. Although research in this area is still in its relative infancy (Smith et al., 2011), several key features have highlighted relaxin-3/RXFP3 systems as an attractive putative target for the treatment of cognitive deficits, and neuropsychiatric disorders, including depression.

**FIGURE 1:**
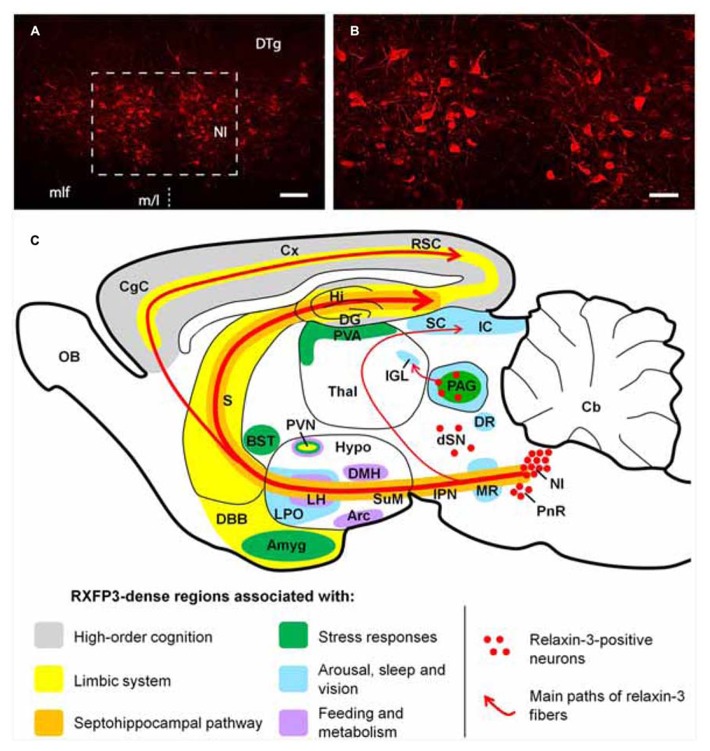
**(A,B)** Low and high magnification micrographs of a coronal section through the mouse NI, displaying neurons positive for relaxin-3-like fluorescent immunoreactivity. The region displayed in **(B)** is outlined in **(A)**. The location of the midline (m/l) is indicated with a dotted line. Anterior-posterior coordinates from bregma, -5.38 mm. Scale bars **A**, 100 μm; **B**, 250 μm. **(C)** Schematic parasagittal representation of the rodent brain, illustrating the ascending relaxin-3 system and the distribution of RXFP3 in regions grouped by function. Amyg, amygdala; Arc, arcuate nucleus; BST, bed nucleus of stria terminalis; Cb, cerebellum; CgC, cingulate cortex; Cx, cerebral cortex; DBB, diagonal band of Broca; DG, dentate gyrus; DMH, dorsomedial nucleus of hypothalamus; DR, dorsal raphé nucleus; dSN, region dorsal to the substantia nigra; DTg, dorsal tegmental nucleus; Hi, hippocampus; Hypo, hypothalamus; IC, inferior colliculus; IGL, intergeniculate leaflet; IPN, interpeduncular nucleus; LH, lateral hypothalamus; LPO, lateral preoptic area; MLF, medial longitudinal fasciculus; MR, median raphé; NI, nucleus incertus; OB, olfactory bulb; PAG, periaqueductal gray; PnR, pontine raphé; PVA, paraventricular thalamic area; PVN, paraventricular hypothalamic nucleus; RSC, retrosplenial cortex; S, septum; SC, super colliculus; SuM, supramammillary nucleus; Thal, thalamus.

Neuroanatomical studies conducted in the rat (Burazin et al., 2002; Tanaka et al., 2005; Ma et al., 2007), mouse (Smith et al., 2010) and macaque (Ma et al., 2009b,c) have revealed that relaxin-3 is mainly expressed within neurons of the pontine *nucleus incertus *(NI;**Goto et al., 2001; Olucha-Bordonau et al., 2003; Ryan et al., 2011), while smaller populations are present in the pontine raphé, periaqueductal gray, and a region dorsal to the substantia nigra (see **Figure 1**). Relaxin-3 containing neurons in these areas innervate a broad range of target forebrain regions rich in RXFP3. NI relaxin-3 neurons are predominately GABAergic (Ma et al., 2007; Cervera-Ferri et al., 2012), and it is likely relaxin-3 signaling confers complimentary inhibitory effects to the primary transmitter, as in cell-based studies RXFP3 activation is linked to G_i/o_ and reduces cAMP accumulation (van der Westhuizen et al., 2007). In recent electrophysiological experiments, however, RXFP3 activation was able to hyperpolarize *or *depolarize presumed RXFP3-positive neurons within the rat intergeniculate leaflet (Blasiak et al., 2013), suggesting the effect of receptor activation or inhibition may vary with the neurochemical phenotype and connectivity of the target neuron, as described for other peptides. RXFP3 activation also stimulates ERK1/2 MAP kinase and other pathways *in vitro* (van der Westhuizen et al., 2010), although related changes in gene expression or precise roles of RXFP3 signaling within distinct neuronal populations *in vivo* remain unknown.

The distribution of relaxin-3-positive axons and RXFP3 mRNA/binding sites within key midbrain, hypothalamic, limbic, and septohippocampal circuits of the rodent and primate brain (Ma et al., 2007, 2009b; Smith et al., 2010) suggests relaxin-3/RXFP3 neural networks represent an “arousal” system that modulates behavioral outputs such as feeding and the responses to stress; and associated neuronal processes including spatial and emotional memory and hippocampal theta rhythm (see **Figure 1**). These actions have been investigated in a number of functional studies in rodents (see Ma et al., 2009a; Smith et al., 2011; Ganella et al., 2012 for review). As numerous neuropsychiatric disorders are either associated with alterations in these processes and behaviors, and/or can be therapeutically treated by drugs which modulate these processes and behaviors (Mazure, 1998; Anand et al., 2005; McGonigle, 2011; Tandon, 2011; Millan et al., 2012), the relaxin-3/RXFP3 system has considerable potential as a novel therapeutic target and warrants further investigation.

## RELAXIN-3/RXFP3 SIGNALING: A NOVEL TARGET FOR THE TREATMENT OF DEPRESSION?

### IS RELAXIN-3 IS AN “AROUSAL” TRANSMITTER?

Wakefulness, along with highly aroused behavioral states such as when an animal is alert, attentive, active, or engaged in exploratory behavior, are mediated by the interactive signaling of a range of “arousal” neurotransmitters (Saper et al., 2005). Several arousal transmitters and their associated neural networks and single or multiple target receptors have been identified, including the monoamines 5-HT, acetylcholine, noradrenaline, and dopamine (Nestler, 1998; Saper et al., 2005; Berridge et al., 2012), and the peptides orexin, melanin-concentrating hormone (MCH) NPY, CRF, and NPS (Hökfelt et al., 2003; Xu et al., 2004; Ishida et al., 2007; Sakurai, 2007; Zee and Manthena, 2007; Bittencourt, 2011). Indeed, it is now widely thought, based particularly on studies using optogenetic control of neural pathways, that selective spatiotemporal recruitment and coordinated activity of various cell type-specific brain circuits may underlie the neural integration of reward, learning, arousal, and feeding.

As mentioned, considerable neuroanatomical evidence suggests relaxin-3 should be thought of as an arousal neurotransmitter. For example, relaxin-3 neurons project to several areas that regulate arousal, such as the midbrain, cortex, thalamus, and limbic and septohippocampal regions, in a similar way as the monoamine and other peptide arousal systems (Ma et al., 2007; Smith et al., 2010, 2011). In fact, the “restricted” localization of relaxin-3 (GABA) neurons and the broadly distributed relaxin-3 projections throughout the brain are remarkably similar to those of the raphé/5-HT (Steinbusch, 1981; Monti and Jantos, 2008; Lesch and Waider, 2012) and locus coeruleus/noradrenaline (Jones et al., 1977; Takagi et al., 1980; Berridge et al., 2012) pathways/networks.

Arousal neurotransmitter systems are extensively interconnected, and relaxin-3 fibers, and RXFP3 are enriched within the pedunculopontine/laterodorsal tegmentum and basal forebrain, periaqueductal gray and lateral hypothalamus; which contain interconnected populations of neurons which produce acetylcholine, dopamine and orexin/MCH, respectively (Saper et al., 2005). Furthermore, along with 5-HT and orexin fibers and receptors (Meyer-Bernstein and Morin, 1996; Marchant et al., 1997; Peyron et al., 1998; Thankachan and Rusak, 2005; Pekala et al., 2011), relaxin-3 fibers/RXFP3 are enriched within the sensory and photic integrative thalamic center, known as the intergeniculate leaflet (Harrington, 1997; Morin, 2013), and application of an RXFP3 agonist can excite (depolarize) NPY neurons within this region (Blasiak et al., 2013), which project to the suprachiasmatic nucleus and promote wakefulness (Shinohara et al., 1993; Thankachan and Rusak, 2005; Zee and Manthena, 2007). Furthermore, rat NI relaxin-3 neurons express the 5-HT_1A_ receptor (and possibly other 5-HT receptors), and chronic 5-HT depletion increased relaxin-3 mRNA in the NI (Miyamoto et al., 2008); while in preliminary electrophysiological studies, bath application of orexin activated rat NI relaxin-3 neurons in a brain slice preparation (Blasiak et al., 2010).

Indeed, arousal and stress transmitter systems, including CRF and orexin peptides and their receptors, have long been implicated in reward and drug seeking behavior (Koob, 2010; Kim et al., 2012) and we recently demonstrated that antagonism of RXFP3 in brain – specifically within the bed nucleus of the stria terminalis – reduced self-administration of alcohol and cue- and stress (yohimbine)-induced relapse in alcohol-preferring iP rats (Ryan et al., 2013b). As monoamines (Nutt et al., 1999; Berridge et al., 2012) and peptides (Nemeroff, 1992; Brundin et al., 2007; McGonigle, 2011) are established or putative targets for the development of antidepressant drugs (Willner et al., 2013), the status of relaxin-3/RXFP3 as a similar and likely interconnected arousal system suggests a similar therapeutic potential.

Abnormal sleep and the disruption of circadian rhythm are common symptoms of the major neurodegenerative diseases (Hastings and Goedert, 2013) and neurological disorders such as depression (Berger et al., 2003), schizophrenia (Van Cauter et al., 1991), and anxiety (Monti and Monti, 2000), and the success of current pharmacological treatments for these diseases appears to be mediated in part through normalizing these symptoms (McClung, 2007). In line with neuroanatomical features, a number of functional studies suggest that relaxin-3 signaling promotes wakefulness. In rats, relaxin-3 mRNA displays a circadian pattern of expression which peaks during the dark/active phase (Banerjee et al., 2005), and intracerebroventricular (icv) infusion of an RXFP3 agonist during the light/inactive phase has been reported to increase locomotor activity (Sutton et al., 2009). These data were partly replicated in mice, in which chronic virally mediated delivery of an RXFP3 agonist into the cerebroventricular system slowed the decline in locomotor activity associated with habituation to a novel environment (Smith et al., 2013a). Mixed background (129S5:B6) relaxin-3 knockout (KO) mice were also hypoactive compared to wildtype littermate controls when placed in novel environments (Smith et al., 2009), and although this phenotype was not reproduced in C57BL/6J backcrossed colonies; during the dark/active phase backcrossed relaxin-3 KO mice traveled less distance on voluntary home-cage running wheels and appeared to spend more time sleeping than wildtype controls (Smith et al., 2012). These data are consistent with a possible regulation of circadian activity by relaxin-3/RXFP3 signaling in the IGL and network-induced changes in SCN activity (Blasiak et al., 2013), a possibility that is currently being explored in both wildtype and gene deletion mouse strains (Hosken et al., 2013).

### RELAXIN-3 NEURONS ARE INVOLVED IN THE RESPONSE TO STRESS

A current view of the stress response is the behavioral and physiological changes generated in the face of, or in anticipation of, a perceived threat. The stress response involves activation of the sympathetic nervous system and recruitment of the hypothalamic-pituitary-adrenal (HPA) axis. When an animal encounters a social, physical or other stressor, these endogenous systems are stimulated and generate a “fight-or-flight” response to manage the “stressful” situation. Acutely, these changes are considered advantageous, but when an organism is subjected to prolonged or chronic stressors, the continuous irregularity in homeostasis is considered detrimental and leads to metabolic and behavioral disturbances (McEwen, 2007). Chronic stress is a well-known trigger for depression in humans, which often involves prolonged over-activation of the HPA axis, resulting in increased circulating glucocorticoids (Mazure, 1998; McEwen, 2007). Since its discovery in 1982 by the late Wylie Vale and others (Bittencourt, 2013), CRF has been shown to play a key role in the stress response *and* in major depression (Nemeroff, 1992; Arborelius et al., 1999; Paez-Pereda et al., 2011). A major source of CRF expression is the parvocellular neurons of the paraventricular hypothalamic nucleus (PVN) that project to the portal circulatory system. In response to stress, CRF is released which triggers the HPA axis by stimulating the release of adrenocorticotropic hormone (ACTH) by the pituitary gland. ACTH binds to receptors in the adrenal gland, which responds by secreting cortisol (corticosterone in rodents). CRF is also expressed within a number of other brain regions including the extended amygdala and the raphé nuclei (Cummings et al., 1983; Morin et al., 1999) and produces a range of extra-pituitary effects via CRF_1_ and CRF_2_ receptors that are broadly expressed throughout the brain (Chalmers et al., 1995; Van Pett et al., 2000).

Relaxin-3 neurons within the rat NI express high levels of CRF_1_ receptor (Bittencourt and Sawchenko, 2000; Tanaka et al., 2005; Ma et al., 2013), and the majority of these neurons are activated (i.e., display increased relaxin-3 mRNA, Fos immunoreactivity and/or depolarization) following a restraint stress or icv injection of CRF (Tanaka et al., 2005; Lenglos et al., 2013; Ma et al., 2013). Relaxin-3 expression in the NI was also increased following a repeated swim stress, and this effect was blocked via pre-administration of the CRF_1_ antagonist, antalarmin (Banerjee et al., 2010). NI neurons are also activated by a range of other stressors, including foot shock, treadmill running, and food deprivation (Ryan et al., 2011), although their impact on relaxin-3 expression has not been assessed. Similarly, the responsiveness of the other relaxin-3 neuron populations has not yet been investigated. The stress-responsiveness of relaxin-3 neurons appears highly conserved, as gene microarray analysis of three-spine stickleback fish revealed that exposure to a predator markedly increased relaxin-3 expression in the brain compared to controls (Sanogo et al., 2011).

Although the precise location and identity of the CRF neurons that innervate relaxin-3 neurons is unknown, the NI receives strong afferent inputs from the CRF-rich lateral and medial preoptic area (Lenglos et al., 2013; Ma et al., 2013), while the close proximity of the NI to the fourth ventricle suggests that volume transfer is also possible (Bittencourt and Sawchenko, 2000). Current data (Lenglos et al., 2013; Ma et al., 2013) and the plasticity of CRF *and* CRF receptor expression (see Dabrowska et al., 2013) suggest the level of CRF innervation and activation of the NI/(relaxin-3) cells may be altered under different physiological and pathological conditions, along with other aspects of their overall phenotype.

In addition to responding to stress, relaxin-3/RXFP3 signaling is able to modulate a variety of stress-related responses. In a recent report, C57B/6J backcrossed relaxin-3 KO mice were reported to display a “subtle decrease” in anxiety-like behavior compared to WT controls (Watanabe et al., 2011b), although a similar phenotype was not observed in a largely parallel study (Smith et al., 2012). In a more relevant set of experiments which highlight the anti-depressant potential of relaxin-3/RXFP3 signaling, icv infusion of a specific RXFP3 agonist reduced anxiety- and depressive-like behavior in rats (Ryan et al., 2013a). These findings have been partly corroborated by an independent study, which observed similar reductions in anxiety-like behavior following icv infusion of relaxin-3 in rats (Nakazawa et al., 2013). These pharmacological effects might be mediated, at least in part, by actions in the amygdala, which is largely responsible for conferring anxiety-related symptoms that are commonly experienced during depression (Holmes et al., 2012). The central and medial amygdala displays some of the highest densities of RXFP3 expression within the rodent brain (Ma et al., 2007; Smith et al., 2010), and injection of a specific RXFP3 agonist into the central amygdala reduced the characteristic freezing fear response displayed by rats when anticipating a foot shock following conventional auditory fear conditioning (Ma et al., 2010).

Relaxin family peptide 3 receptor expression is also highly enriched within the PVN (Ma et al., 2007; Smith et al., 2010), and icv injection of relaxin-3 in rats increased CRF and c-*fos* mRNA within the PVN and increased plasma ACTH, indicative of HPA axis activation (Watanabe et al., 2011a). These findings suggest that although the net sum of behavioral responses following “global” (or intra-amygdala) RXFP3 activation appears to be anxiolytic/antidepressant in nature (Nakazawa et al., 2013; Ryan et al., 2013a), RXFP3 signaling can in fact either promote or attenuate different aspects of the stress response, depending on the brain region modulated. This feature is shared with several other neuropeptides. For example, rodent studies have demonstrated that orexin and galanin signaling can either increase or decrease anxiety-like behavior, depending on the brain region(s) targeted (Bing et al., 1993; Möller et al., 1999; Lungwitz et al., 2012), while icv administration of NPS has been shown to decrease anxiety while increasing HPA axis activity (Xu et al., 2004; Smith et al., 2006). NPY (possibly from the arcuate nucleus) can activate the HPA axis via NPY Y1 receptors expressed on PVN CRF neurons (Albers et al., 1990; Dimitrov et al., 2007); but icv administration of NPY and a specific Y1 agonist inhibits fear behavior during contextual fear conditioning (Lach and de Lima, 2013).

High densities of relaxin-3-positive fibers and RXFP3 mRNA/binding sites are also present within several other brain structures that contribute to the central stress response and have been implicated in the etiology of anxiety and depression (Ma et al., 2007; Smith et al., 2010), including the:**(i) *dorsal raphé*, which contains stress-responsive 5-HT neurons that are critical for determining depression susceptibility and recovery (Zhang et al., 2012; Challis et al., 2013); (ii) *hippocampus*, which expresses high densities of glucocorticoid receptors and often displays reduced volume and neurogenesis and impaired function in depressed patients (Manji et al., 2003; Videbech and Ravnkilde, 2004; Willner et al., 2013); (iii) *periaqueductal* *gray*, which is involved in fear behavior and associated autonomic responses (Vianna et al., 2001), and which contains relaxin-3 neurons positive for CRF_1/2_ immunoreactivity (Blasiak et al., 2013); (iv) *bed nucleus of the stria terminalis*, which constitutes a stress integration center that contains CRF-expressing and other peptide containing GABA/glutamate neurons, which strongly influence the PVN and are reportedly dysfunctional in several psychiatric disorders, including depression, anxiety-disorders, and addiction (Dunn, 1987; Walker et al., 2009; Koob, 2010; Lebow et al., 2012; Crestani et al., 2013; Zheng and Rinaman, 2013); (v) *medial preoptic area*, in which neurons also express high levels of CRF and strongly project to and influence the PVN (Marson and Foley, 2004; Lenglos et al., 2013); (vi) *lateral habenula*, a key structure mediating the response to emotionally negative states (Willner et al., 2013), in which neuron activity was shown recently to be regulated by levels of β-CaMK II expression and to be sufficient to either induce or alleviate depressive-like symptoms in rodents, depending on whether these neurons were activated or inhibited, respectively (Li et al., 2013); (vii) *anterior cingulate cortex*, which acts to stabilize emotional responses via inhibitory projections to the amygdala that are often reduced in depressed patients (Anand et al., 2005; Willner et al., 2013) and; (viii) *medial prefrontal cortex*, which is dysfunctional in depressed patients and strongly projects to the PVN and amygdala to suppress behavioral responses to stress (Espejo and Minano, 1999). The medial prefrontal cortex is of additional interest, as it forms a main source of afferent input into the NI (Goto et al., 2001). A recent study has also demonstrated that stimulation of CRF_1_ positive NI neurons that project to the medial prefrontal cortex (either electrophysiologically or via administration of CRF) act to inhibit this region, while electrical or CRF-mediated stimulation of the whole NI impaired long term potentiation within the hippocampo-prelimbic medial prefrontal cortical pathway (Farooq et al., 2013).

### RELAXIN-3 NEURONS MODULATE HIPPOCAMPAL ACTIVITY

A key feature of hippocampal function is a state of synchronous neuronal firing at theta rhythm (4–10 Hz in humans), which is required for the hippocampus to mediate its important roles in memory formation and retrieval, spatial navigation, and rapid eye movement (REM) sleep (Vertes and Kocsis, 1997). Hippocampal function is disrupted by elevated circulating glucocorticoids during chronic stress, which can contribute to the cognitive deficits seen in depression (Murphy et al., 2001; Clark et al., 2009). Furthermore, a common hallmark of depression is stress-related increases in REM sleep (Kimura et al., 2010), which is robustly reduced to normal levels following antidepressant treatment (Argyropoulos and Wilson, 2005), an effect partly mediated by 5-HT signaling (Adrien, 2002). In light of the critical role that hippocampal theta rhythm plays in normal neurological function and its propensity for disruption in disease states, it is not surprising that almost all currently available anxiolytic and pro-cognitive drugs alter hippocampal theta rhythm (McNaughton and Gray, 2000). It has in fact been suggested that this feature can be used as an “output” for screening the potential effectiveness of new psychoactive drugs (McNaughton et al., 2007).

The ability of ascending brainstem nuclei such as the *reticularis pontis oralis* (RPO) and median raphé to modulate hippocampal theta rhythm is well established. These functions are mediated not only by projections to the hippocampus, but also via innervation of several “nodes” of the septohippocampal system such as the interpeduncular nucleus (IPN), supramammillary nucleus, posterior hypothalamus, and medial septum (Vertes and Kocsis, 1997). In particular, the medial septum has been termed the hippocampal theta rhythm “pace-maker” and contains populations of cholinergic and GABAergic neurons which provide alternating synchronous excitatory/inhibitory input to reciprocally connected hippocampal neurons (Vertes and Kocsis, 1997; Wang, 2002; Hangya et al., 2009). The NI sits adjacent to, and is strongly interconnected with, the RPO, median raphé and IPN, and efferent relaxin-3-positive projections innervate the hippocampus and the major nodes of the septohippocampal pathway (Ma et al., 2007; Teruel-Marti et al., 2008; Smith et al., 2010; Cervera-Ferri et al., 2012), including the medial septum which displays a high density of relaxin-3 immunoreactive fibers and terminals which make synaptic contacts with hippocampal-projecting cholinergic and GABAergic neurons in the rat (Olucha-Bordonau et al., 2012).

Functional studies have confirmed the regulation of hippocampal theta rhythm by the NI. In anesthetized rats, electrical stimulation of the NI induced hippocampal theta rhythm, whereas electrolytic lesion of the NI blocked the ability of the RPO to generate hippocampal theta rhythm (Nunez et al., 2006; Teruel-Marti et al., 2008). In conscious rats with electrolytic lesions of the NI, theta-dependent behaviors are impaired such as the acquisition of fear extinction in a contextual auditory conditioned fear paradigm (Pereira et al., 2013). Simultaneous recording of hippocampal and NI field potentials (Cervera-Ferri et al., 2011) and electrophysiological recording of NI neurons (Ma et al., 2013) have also revealed that these two structures are “theta-synchronized” and individual neurons display coherent firing. Although it is likely that these actions are primarily conferred by GABA (or to a much lesser extent, glutamate) transmission of these septohippocampal-projecting NI neurons (Ma et al., 2007; Cervera-Ferri et al., 2012), relaxin-3/RXFP3 signaling nonetheless appears capable of contributing to this functional effect. Our laboratory has shown that local infusion of an RXFP3 agonist into the medial septum of anesthetized rats promotes hippocampal theta rhythm, while medial septum infusion of an RXFP3 antagonist in conscious rats inhibits hippocampal theta and theta-dependent spatial memory measured in a spontaneous alternation task (Ma et al., 2009a).

## RELEVANCE OF RELAXIN-3/RXFP3 SIGNALING TO SOCIAL BEHAVIOR AND AUTISM?

In rodents, social behavior is highly dependent upon three aspects of brain function: (i) *arousal*, which is required for motivation to engage in social contact, and mediates appropriate mood responses (Crawley et al., 1981); (ii) *stress responses*, which regulate levels of social withdrawal/anxiety (File and Seth, 2003) and; (iii) *exploration and social recognition*, which is associated with hippocampal theta rhythm activity (Maaswinkel et al., 1997). Notably, relaxin-3 has been demonstrated to modulate all of these behavioral aspects.

Abnormal social behavior is associated with depression and is a key symptom of ASD (Millan et al., 2012; Bishop-Fitzpatrick et al., 2013). Human imaging studies indicate that autism is often characterized by structural abnormalities in limbic structures such as the hippocampus (Haznedar et al., 2000; Ohnishi et al., 2000), which according to post-mortem studies consists of principal neurons that are smaller in size and are more densely packed (Bauman and Kemper, 2005). The amygdala is another major limbic structure that has been the focus of many human (van Elst et al., 2000) and animal (Amaral et al., 2003) studies of social aggression, and in rodent models of autism, hyperexcitability and enhanced long term potentiation in lateral amygdala neurons has been reported (Lin et al., 2013). Reduced activity of the anterior cingulate cortex has been observed in human autistic patients, which is correlated with deficits in attention and executive control (Fan et al., 2012). The PVN is another major limbic structure relevant to autism partly due to the presence of oxytocin neurons, which are crucial for mother-infant bonding (Mogi et al., 2010) and promote social interaction (Lukas et al., 2011). Autism is associated with loss of PVN oxytocin neurons (McNamara et al., 2008), and oxytocin is displaying considerable promise in clinical treatment of this disorder (Yamasue et al., 2012). The PVN also contains neurons that express vasopressin, which reciprocally interact with oxytocin neurons and strongly influence social behaviors such as aggression (Caldwell et al., 2008), suggesting similar therapeutic potential (Ring, 2011; Lukas and Neumann, 2013).

Relaxin-3/RXFP3 systems are well placed to modulate social behavior and other symptoms of ASD due to their presence throughout the limbic hippocampus, amygdala, anterior cingulate cortex, and PVN. Particularly intriguing, however, is the strong link between relaxin-3 and oxytocin. Oxytocin receptors are expressed within the rat and mouse NI (Vaccari et al., 1998; Yoshida et al., 2009), and microarray/peptidomics analysis revealed that the most striking neurochemical change that occurred within the rat hypothalamus following acute icv infusion of relaxin-3 and resultant activation of RXFP3 (and RXFP1) was a large (>10-fold) upregulation of oxytocin (Nakazawa et al., 2013). In contrast, chronic hypothalamic RXFP3 signaling resulted in an opposite effect, as viral-mediated hypothalamic delivery of an RXFP3 agonist for 3 months reduced hypothalamic oxytocin mRNA by ~50% (Ganella et al., 2013a). Whether some or all oxytocin neurons express RXFP3 or whether these effects are mediated in part or in full by indirect actions, remains to be determined experimentally. Similarly, vasopressin neurons may also be targeted by RXFP3 signaling (Ganella et al., 2013a).

Despite the potential for a role of relaxin-3/RXFP3 signaling in aspects of social behavior, only a single functional study has thus far been reported, which observed that compared to wildtype littermate controls, female 129S5:B6 mixed background relaxin-3 KO mice engaged in fewer encounters with a novel mouse in a social interaction test (Smith et al., 2009). Therefore, further studies including those that test the therapeutic potential of RXFP3 agonists in validated rodent models of major ASD symptoms are required. These might also include assessment of aggressive behavior, with the presence of RXFP3 in brain “defensive centers” such as the amygdala, PAG, and ventromedial hypothalamus (see Future Studies of Relaxin-3/RXFP3 System).

## RELAXIN-3/RXFP3 CONTROL OF FEEDING AND RELEVANCE FOR EATING DISORDERS?

It is generally accepted that obesity has rapidly reached epidemic proportions, but is also one of the leading preventable causes of death worldwide. Notably, there is evidence that obesity associated metabolic signals markedly increase the odds of developing depression; and depressed mood not only impairs motivation, quality of life and overall functioning, but also further increases the risks of complications associated with obesity (Hryhorczuk et al., 2013). Therefore, curbing the global growth in obesity and associated health problems, and demands on public healthcare, is a major challenge which offers huge economic reward for agencies that develop effective treatments (Kopelman, 2000; Carter et al., 2012; Roux and Donaldson, 2012; Adan, 2013). Conversely, a smaller but important niche exists for the development of orexigenic agents to treat symptoms of decreased appetite and/or cachexia associated with cancer and its treatment, immune deficiency, and anorexia nervosa (Sodersten et al., 2006).

RXFP3 is present in several hypothalamic feeding centers in rat brain (Kishi and Elmquist, 2005) including the PVN (Liu et al., 2003), lateral hypothalamus, arcuate, and dorsomedial nuclei (Sutton et al., 2004; Ma et al., 2007). These data prompted a series of pharmacological studies which consistently demonstrated that relaxin-3 and selective RXFP3 agonist peptides are potently orexigenic in rats following acute delivery into the lateral cerebral ventricle (Liu et al., 2005, 2009; McGowan et al., 2005; Sutton et al., 2009; Shabanpoor et al., 2012; Hossain et al., 2013) or various hypothalamic regions (McGowan et al., 2007). Chronic delivery of RXFP3 agonists via repeated intra-PVN injection (McGowan et al., 2006), osmotic minipump (icv) infusion (Hida et al., 2006; Sutton et al., 2009), or viral constructs injected into the PVN (Ganella et al., 2013a) also reliably increase food consumption and bodyweight, and result in metabolic changes such as increased plasma levels of leptin, insulin, and adiponectin, and decreased plasma levels of growth hormone and thyroid stimulating hormone. Co-administration of RXFP3 antagonists are able to prevent the increases in feeding induced by acute RXFP3 agonist injections (Kuei et al., 2007; Haugaard-Jonsson et al., 2008), but a significant reduction in feeding behavior produced by acute blockade of endogenous relaxin-3/RXFP3 signaling in satiated *and* food restricted rats is yet to be reported; suggesting a graded impact on this heavily regulated homeostatic behavior. Furthermore, relaxin-3 KO mice (C57BL/6J background) do not display any overt differences in feeding or bodyweight under normal housing and dietary conditions (Watanabe et al., 2011b; Smith et al., 2012), despite an earlier report that mixed (129S5:B6) background relaxin-3 KO mice fed on a diet with higher than normal (moderate) fat content were largely resistant to the obesity observed in WT controls (Sutton et al., 2009). Clearly this is an important area for further research in normal and other suitable transgenic mice. A recent study suggested that relaxin-3/RXFP3 signaling may be more important under specific physiological conditions, as in stressed female rats with intermittent access to palatable liquid food, relaxin-3 expression in the NI was increased in food restricted versus *ad libitum* fed animals (Lenglos et al., 2013).

Increased feeding is a common side effect of antipsychotic medications (Theisen et al., 2003), and acute atypical (clozapine) and typical (chlorpromazine and fluphenazine) antipsychotic treatments increased the number of Fos-positive cells in the rat NI (Rajkumar et al., 2013). On this basis, it was hypothesized that increased NI activation may be partly responsible for the antipsychotic drug induced increase in feeding behavior, which if correct, would suggest that relaxin-3/RXFP3 signaling might also play a role. Further evidence supporting this theory comes from a gene association study, in which >400 schizophrenia patients undergoing treatment with antipsychotic medications were assessed, many of whom displayed co-morbid metabolic syndromes (Munro et al., 2012). Interestingly, a polymorphism within the RXFP3 gene was significantly associated with obesity, while one polymorphism in the relaxin-3 gene and two in the RXFP3 gene were significantly associated with hypercholesterolemia.

In another gene association study, members of a Puerto Rican family with schizophrenia had a mutation within a chromosome 5p locus, which had earlier been identified in similar studies of familial schizophrenia-like symptoms (Bespalova et al., 2005). This locus contains the RXFP3 gene, and although sequencing of the coding region and proximal promoter did not reveal functionally significant variants, further upstream or downstream promoter regions were not assessed. Antipsychotics block dopamine D2 receptors and are the primary therapy for psychotic, *positive* symptoms (hallucinations/delusions) of schizophrenia (Tandon, 2011; Castle et al., 2013). It is possible, however, that modulation of endogenous relaxin-3/RXFP3 signaling might reduce the severity of the *negative* affective symptoms and cognitive deficits displayed in schizophrenic patients. These putative roles might be mediated via actions within limbic structures to modulate relevant neural circuits that regulate theta and other frequency brain oscillations, to enhance attention, working, and episodic memory (Ma et al., 2009a; Millan et al., 2012). However, experimental evidence in support of this speculation is yet to be gathered.

Overall, given the enormity of the obesity epidemic and associated health problems and the lack of understanding of, and effective pharmacological therapies for, eating disorders such as anorexia nervosa, there is a strong justification for further studies that involve chronic manipulation of RXFP3 signaling to assess feeding, metabolism, and body weight.

## FUTURE STUDIES OF THE RELAXIN-3/RXFP3 SYSTEM

Considerable experimental evidence obtained over the last decade suggests that endogenous relaxin-3/RXFP3 signaling promotes arousal and contributes to the central response to stress, and the highly conserved nature of this peptide/receptor system suggests it plays important biological roles. Current data suggest that drugs which act to increase relaxin-3/RXFP3 signaling are likely to have therapeutic/beneficial effects in a range of clinical conditions. Like many other complex neuromodulatory (peptide) systems, however, receptor modulation in different brain regions may confer differential effects; and in a therapeutic context, increased brain RXFP3 activation may produce both beneficial *and* “undesirable” effects. With RXFP3 agonists, in some disorders these may include increased HPA axis activity (Watanabe et al., 2011a) and bodyweight gain (McGowan et al., 2005; Ganella et al., 2012; Lenglos et al., 2013); while with RXFP3 antagonists these may include decreased arousal and motivation. Therefore, characterizing precise direct and indirect actions of relaxin-3/RXFP3 signaling within the major RXFP3-rich regions of the rodent brain remains an important long term goal. Similarly, neurons in the relaxin-3 rich NI express a large array of receptors for transmitters, and monoamine and peptide modulators (Blasiak et al., 2010; Ryan et al., 2011; Ma et al., 2013), and it will be important to carefully assess how these signals are integrated by the NI relaxin-3 system.

Studies which have centrally administered RXFP3 agonists have mainly employed the icv route, and although it is often assumed that peptides are able to access receptors throughout the whole brain (Bittencourt and Sawchenko, 2000), recent studies in our laboratory using fluorophore-conjugated relaxin family peptides suggest that periventricular regions such as the PVN may be exposed to higher concentrations of peptide (Chan et al., 2013). Although RXFP3 agonists or antagonists have been locally infused into the bed nucleus of the stria terminalis (Ryan et al., 2013b), central amygdala (Ma et al., 2010), medial septum (Ma et al., 2009a), and hypothalamic nuclei (McGowan et al., 2007) of rodents, in connection with actions on reward, fear, spatial memory, and feeding, respectively; many other RXFP3-rich brain regions including those distal to the ventricular system remain to be targeted, including the median raphé, superior and inferior colliculus, intergeniculate leaflet, IPN, supramammillary nucleus, diagonal band of Broca, fields within the dorsal and ventral hippocampus, and the retrosplenial and cingulate cortices (see **Figure 1**). Intranasal delivery may be a viable alternate route of peptide administration based on recent studies with insulin, oxytocin/vasopressin and NPS (e.g., Ionescu et al., 2012), but ultimately, characterization of the net effects of activating RXFP3 throughout the brain is required, using highly stable peptides or small synthetic molecules that cross the blood–brain barrier (Bathgate et al., 2013b) and can be administered systemically.

In addition to characterizing the function of relaxin-3/RXFP3 at a regional level, it is crucial to characterize the populations of neurons that express RXFP3 within each nucleus/region, and whether they are stimulated or inhibited following RXFP3 activation. Such functional data will provide valuable insights into the mechanisms of relaxin-3 action, but to date, this has only been partially achieved in the intergeniculate leaflet (Blasiak et al., 2013). Based on equivalent studies of similar systems such as the orexins, such features may be complicated, despite the relative simplicity of the one ligand/one receptor, relaxin-3/RXFP3 system.

In the context of arousal, an RXFP3-rich area of particular interest is the lateral hypothalamus (Ma et al., 2007; Smith et al., 2010). If it is assumed that RXFP3 activation *inhibits* receptor-positive neurons, then it is possible that relaxin-3/RXFP3 may promote arousal by directly inhibiting neurons which express MCH, which act to inhibit arousal (Saito and Nagasaki, 2008). Alternatively, activation of RXFP3 expressed on GABAergic interneurons which project to and inhibit orexin/dynorphin/(neurotensin) neurons in the area (Alam et al., 2005; Burt et al., 2011; Furutani et al., 2013), may indirectly disinhibit these neurons, increasing the activity of these arousal-promoting networks. If, however, RXFP3 signaling directly *stimulates* specific target neurons, these scenarios could be reversed. Similar hypothetical circuits can be conceived involving sleep active neurons that express galanin in the ventrolateral preoptic area (Gaus et al., 2002), 5-HT and non 5-HT neurons in the dorsal and median raphé (Morin and Meyer-Bernstein, 1999; Kirby et al., 2000; Kocsis et al., 2006), and a host of other systems throughout the brain (Smith et al., 2013b). Traditional immunohistochemical approaches to achieving this goal have been hampered, however, as sufficiently sensitive and specific antisera for RXFP3 are currently unavailable. An alternative approach has observed relaxin-3-positive fibers in the rat medial septum terminating on neurons expressing choline acetyltransferase, parvalbumin, and glutamate decarboxylase (Olucha-Bordonau et al., 2012); but this “indirect” method is labor intensive and future studies would benefit from the development of an RXFP3 antibody, or transgenic mice which express a reporter gene under the control of the RXFP3 promoter (e.g., Chee et al., 2013).

Acute icv infusion of an RXFP3 agonist decreased the time rats spent immobile in the Porsolt forced swim test (Ryan et al., 2013a), which is used to test for putative antidepressant drug action. However, more recently this measure of “depressive-like” behavior has been described as having poor predictive, face, and construct validity (Nestler and Hyman, 2010), particularly as such changes in behavior are evident in rodents following acute administration of SSRIs, while these drugs require chronic administration over weeks in humans before therapeutic effects are observed. It is also possible that the Porsolt paradigm, which was developed to test drugs that target monoamine systems, may not be optimal for assessing drugs that target neuropeptide receptors. Therefore, it will be important to test the antidepressant potential of acute and chronic delivery of RXFP3 agonists against behavioral measures such as anhedonia and aberrant reward-associated perception, and memory in additional validated rodent models of depression, such as the chronic unpredictable mild stress, chronic social defeat, and chronic methamphetamine withdrawal models (Nestler and Hyman, 2010; Russo and Nestler, 2013) and/or assess effects on brain activity patterns (McNaughton and Gray, 2000).

Similarly, it will be of interest to assess whether RXFP3 agonists (or antagonists) can improve social behavior in one or more of the rodent models of ASD, such as the commonly used BTBR (Silverman et al., 2010) and transgenic mouse strains (Peca et al., 2011). Determining whether RXFP3 antagonists are protective against the obesity and metabolic syndromes induced by high fat diets in rodents is also a logical and important goal (Panchal and Brown, 2011; Ganella et al., 2012).

These studies would benefit greatly from the development of small molecule RXFP3 agonists and antagonists with a stable *in vivo* half-life that can cross the blood–brain barrier, and hence could be administered peripherally. Such compounds would penetrate the brain more evenly and in a manner more closely resembling the method that would eventually be adopted in humans, rather than preferentially accessing regions near the ventricular system, which occurs following icv infusions. Peripheral delivery methods also circumvent the need for surgical implantation of indwelling guide cannulae in experimental studies. The development of such compounds has not been reported, however, despite initial efforts by some groups (e.g., Alvarez-Jaimes et al., 2012).

In the meantime, further experimental studies are likely to benefit from recently developed and novel methods to manipulate the relaxin-3/RXFP3 system. For example, the RXFP3 agonist “R3/I5” has been successfully delivered chronically into the PVN of rats using an adeno-associated viral construct (Ganella et al., 2013a), which improves upon previous studies which relied on repeated injections (McGowan et al., 2006) or osmotic minipump infusions of exogenous peptide (Hida et al., 2006; Sutton et al., 2009), which are stressful and invasive techniques that can potentially alter behavior. The development and study of conditional rxfp3 KO mice in which RXFP3 protein could be deleted either globally or within specific brain regions in adult mice would not only help characterize the regional role of endogenous relaxin-3/RXFP3 signaling, but should also prevent the “masking” of phenotypes which may occur due to developmental compensation in life-long relaxin-3 KO mice (Smith et al., 2012).

The clustered/restricted distribution of relaxin-3 neurons within the NI readily enables targeting of these neurons with injected viral constructs (Callander et al., 2012), which could be used to drive the expression of virally encoded genes of interest under the control of the relaxin-3 promoter (Tanaka et al., 2009). Cell type-specific expression of light-gated ion channels has become a powerful resource for the anatomical and functional deconstruction of neuronal networks and allows the structural dynamics and electrical activity of genetically defined neurons to be manipulated and analyzed on the millisecond timescale (Zhang et al., 2010; Yizhar et al., 2011a; Kalmbach et al., 2012). The overall function of relaxin-3 NI neurons could be similarly assessed via targeted expression of channelrhodopsins and related functional measures. Similarly, the expression in NI GABA/relaxin-3 expressing neurons of excitatory and inhibitory “Designer Receptors Exclusively Activated by Designer Drugs” (DREADDs; Nawaratne et al., 2008; Sasaki et al., 2011; Farrella and Roth, 2013; Wess et al., 2013) will allow the effects of acute and chronic activation/inhibition of these neurons on brain circuit activity and behavior to be conveniently studied in freely moving animals. These studies will be important in delineating whether in the NI, it is relaxin-3 or GABA signaling or GABA signaling specifically associated with the relaxin-3-expressing neurons that is primarily linked to effects on brain network activity and changes in behavior (see e.g., GABA/AgRP neurons in the arcuate nucleus in control of feeding (Atasoy et al., 2012; Liu et al., 2013).

For effective drug development in the future, the definition and characterization of depression and antidepressant drug treatment effects, currently based heavily on symptomatic criteria, needs to be improved, so that greater emphasis is placed on the underlying dysfunction at the circuit, neuron, and transmitter level (see Millan et al., 2012; Willner et al., 2013). In this regard, characterizing the potential involvement of novel transmitter systems such as relaxin-3 in the etiology of depression will be of interest. Although relaxin-3 and RXFP3 are genetically highly conserved between rodents and humans, more experiments are needed to demonstrate conserved functions of these signaling networks. The anatomical distribution of relaxin-3 and RXFP3 in non-human primate brain is very similar to that observed in rat and mouse (Ma et al., 2009b,c); and so “select” studies in non-human primates should be informative (Willard and Shively, 2012). Further studies of any potential involvement of relaxin-3 in the etiology of neurological or psychiatric diseases are also warranted (*c.f.* Lin and Sibille, 2013). For example, in addition to comprehensive searches for polymorphisms in the relaxin-3 or RXFP3 genes that might result in altered neurotransmission and affective behavior; once suitably validated assays for human relaxin-3 peptide and/or RXFP3 protein levels are available, studies to determine whether these are altered in patients who suffer from depression and other mental disorders could be completed, as potential markers for dysregulation of relaxin-3/RXFP3 related signaling. Any such findings would, based on prior experience with other peptide-receptor systems such as NPS and PACAP (Pape et al., 2010; Ressler et al., 2011), provide a significant stimulus to this relatively new area of research.

Finally, there are clear signs in the academic literature and emerging from government agencies and Pharma that the field of psychiatric disease research is entering a new era in relation to better understanding and improved drug and environmental-based treatments. This involves an emphasis on analyzing the neural circuitry that causes these brain diseases, rather than a reliance on more “isolated” conventional neurotransmitter and receptor based studies or isolated gene-based studies (Millan et al., 2012; Abbott, 2013; Insel et al., 2013a,b). Thus, newly identified signaling systems like relaxin-3/RXFP3 will need to be studied in the context of regulatory impacts on key neural circuits under physiological and pathological conditions in human (patient-relevant) and industry-validated experimental models, and demonstrate genuine efficacy to restore the required balance of excitatory/inhibitory transmission in one or more diseases.

However, given the relative paucity of new therapeutic drug discoveries in the field over the last several decades using “older style” techniques, this recent realization and redirection in psychiatric disease research in some way removes any disadvantage a “new, little investigated” system such as this might have over other more exhaustively explored systems. Certainly, based on what is known regarding the anatomical distribution of relaxin-3/RXFP3 networks and the prominent effects they can demonstrate on fundamental processes (such as coherent neural firing in the “septohippocampal system” and associated limbic circuits (Farooq et al., 2013; Ma et al., 2013) and effects on circadian activity related circuits (Smith et al., 2012; Blasiak et al., 2013), there is reason for optimism regarding its ability to be relevant therapeutically and to attract the attention of major Pharma.

## CONCLUSION

The study of neuropeptide-receptor systems is a key area of neuropsychopharmacology research and has revealed the involvement of several peptide systems in mental illnesses, in addition to identifying novel targets for their treatment. Relaxin-3 is a highly conserved neuropeptide in mammalian brain. Relaxin-3 neurons located in the midbrain and pons, innervate a broad range of RXFP3-rich circuits (hypothalamic, septohippocampal, and limbic) to modify stress, arousal, and other modalities that are often dysfunctional in neuropsychiatric diseases. Therefore, further elucidating the full array of relaxin-3/RXFP3 network effects under normal and pathological conditions represents an important and promising research goal, which may eventually help meet the challenges and opportunities for improving the symptomatic treatment of sufferers of conditions such as anxiety and major depression, and the social and cognitive deficits in neurodevelopmental, and degenerative disorders, by restoring the required balance of excitatory/inhibitory transmission within the appropriate neural circuits.

## Conflict of Interest Statement

The authors declare that the research was conducted in the absence of any commercial or financial relationships that could be construed as a potential conflict of interest.

## References

[B1] Abbott A. (2013). Novartis reboots brain division. *Nature* 502 153–154 10.1038/502153a 24108029

[B2] Adan R. A. H. (2013). Mechanisms underlying current and future anti-obesity drugs. *Trends Neurosci.* 36 133–140 10.1016/j.tins.2012.12.001 23312373

[B3] Adrien J. (2002). Neurobiological basis for the relation between sleep and depression. *Sleep Med. Rev.* 6 341–351 12531125

[B4] Alam M. N. Kumar S. Bashir T. Suntsova N. Methippara M. M. Szymusiak R. (2005). GABA-mediated control of hypocretin- but not melanin-concentrating hormone-immunoreactive neurones during sleep in rats. *J. Physiol. (Lond.)* 563 569–582 10.1113/jphysiol.2004.076927 15613374 PMC1665577

[B5] Albers H. E. Ottenweller J. E. Liou S. Y. Lumpkin M. D. Anderson E. R. (1990). Neuropeptide Y in the hypothalamus: effect on corticosterone and single-unit activity. *Am. J. Physiol.* 258 R376–R382 2309931 10.1152/ajpregu.1990.258.2.R376

[B6] Alvarez-Jaimes L. Sutton S. W. Nepomuceno D. Motley S. T. Cik M. Stocking E. (2012). In vitro pharmacological characterization of RXFP3 allosterism: an example of probe dependency. *PLoS ONE* 7:e30792 10.1371/journal.pone.0030792 PMC3274524 22347403

[B7] Amaral D. G. Bauman M. D. Capitanio J. P. Lavenex P. Mason W. A. Mauldin-Jourdain M. L. (2003). The amygdala: is it an essential component of the neural network for social cognition? *Neuropsychologia* 41 517–522 10.1016/S0028-3932(02)00310-X 12559167

[B8] Anand A. Li Y. Wang Y. Wu J. Gao S. Bukhari L. (2005). Activity and connectivity of brain mood regulating circuit in depression: a functional magnetic resonance study. *Biol. Psychiatry* 57 1079–1088 10.1016/j.biopsych.2005.02.021 15866546

[B9] Antonijevic I. A. Murck H. Bohlhalter S. Frieboes R. M. Holsboer F. Steiger A. (2000). Neuropeptide Y promotes sleep and inhibits ACTH and cortisol release in young men. *Neuropharmacology* 39 1474–1481 10.1016/S0028-3908(00)00057-5 10818263

[B10] Arborelius L. Owens M. J. Plotsky P. M. Nemeroff C. B. (1999). The role of corticotropin-releasing factor in depression and anxiety disorders. *J. Endocrinol.* 160 1–12 10.1677/joe.0.1600001 9854171

[B11] Argyropoulos S. V. Wilson S. J. (2005). Sleep disturbances in depression and the effects of antidepressants. *Int. Rev. Psychiatry* 17 237–245 10.1080/09540260500104458 16194795

[B12] Atasoy D. Betley J. N. Su H. H. Sternson S. M. (2012). Deconstruction of a neural circuit for hunger. *Nature* 488 172–177 10.1038/nature11270 22801496 PMC3416931

[B13] Bailey J. E. Papadopoulos A. Diaper A. Phillips S. Schmidt M. van der Ark P. (2011). Preliminary evidence of anxiolytic effects of the CRF1 receptor antagonist R317573 in the 7.5% CO2 proof-of-concept experimental model of human anxiety. *J. Psychopharmacol.* 25 1199–1206 10.1177/0269881111400650 21555331

[B14] Banerjee A. Ma S. Ortinau S. Smith C. M. Layfield S. Burazin T. C. D. (2005). Relaxin-3 neurons in the nucleus incertus – projection patterns, response to swim stress and relaxin-3 neuronal signalling. *Soc. Neurosci. Abstr.* 35 59.57

[B15] Banerjee A. Shen P.-J. Ma S. Bathgate R. A. D. Gundlach A. L. (2010). Swim stress excitation of nucleus incertus and rapid induction of relaxin-3 expression via CRF1 activation. *Neuropharmacology* 58 145–155 10.1016/j.neuropharm.2009.06.019 19560474

[B16] Bathgate R. A. D. Halls M. L. van der Westhuizen E. T. Callander G. E. Kocan M. Summers R. J. (2013a). Relaxin family peptides and their receptors. *Physiol. Rev.* 93 405–480 10.1152/physrev.00001.2012 23303914

[B17] Bathgate R. A. D. Oh M. H. Ling W. J. Kaas Q. Hossain M. A. Gooley P. R. (2013b). Elucidation of relaxin-3 binding interactions in the extracellular loops of RXFP3. *Front. Endocrinol. (Lausanne) * 4:13 10.3389/fendo.2013.00013 PMC3579193 23440673

[B18] Bathgate R. A. D. Ivell R. Sanborn B. M. Sherwood O. D. Summers R. J. (2006). International Union of Pharmacology LVII: recommendations for the nomenclature of receptors for relaxin family peptides. *Pharmacol. Rev.* 58 7–31 10.1124/pr.58.1.9 16507880

[B19] Bathgate R. A. D. Samuel C. S. Burazin T. C. D. Layfield S. Claasz A. A. Reytomas I. G. (2002). Human relaxin gene 3 (H3) and the equivalent mouse relaxin (M3) gene. Novel members of the relaxin peptide family.* J. Biol. Chem.* 277 1148–1157 10.1074/jbc.M107882200 11689565

[B20] Bauman M. L. Kemper T. L. (2005). Neuroanatomic observations of the brain in autism: a review and future directions. *Int. J. Dev. Neurosci.* 23 183–187 10.1016/j.ijdevneu.2004.09.006 15749244

[B21] Berger M. Van Calker D. Riemann D. (2003). Sleep and manipulations of the sleep-wake rhythm in depression. *Acta Psychiatr. Scand.* 108 83–91 10.1034/j.1600-0447.108.s418.17.x 12956821

[B22] Berridge C. W. Schmeichel B. E. España R. A. (2012). Noradrenergic modulation of wakefulness/arousal. *Sleep Med. Rev.* 16 187–197 10.1016/j.smrv.2011.12.003 22296742 PMC3278579

[B23] Bespalova I. N. Angelo G. W. Durner M. Smith C. J. Siever L. J. Buxbaum J. D. (2005). Fine mapping of the 5p13 locus linked to schizophrenia and schizotypal personality disorder in a Puerto Rican family. *Psychiatry Genet.* 15 205–210 10.1097/00041444-200509000-00012 16094256

[B24] Bing O. Möller C. Engel J. A. Soderpalm B. Heilig M. (1993). Anxiolytic-like action of centrally administered galanin. *Neurosci. Lett.* 164 17–20 10.1016/0304-3940(93)90846-D 7512244

[B25] Binneman B. Feltner D. Kolluri S. Shi Y. Qiu R. Stiger T. (2008). A 6-week randomized, placebo-controlled trial of CP-316,311 (a selective CRH1 antagonist) in the treatment of major depression. *Am. J. Psychiatry* 165 617–620 10.1176/appi.ajp.2008.07071199 18413705

[B26] Bishop-Fitzpatrick L. Minshew N. J. Eack S. M. (2013). A systematic review of psychosocial interventions for adults with autism spectrum disorders. *J. Autism Dev. Disord.* 43 687–694 10.1007/s10803-012-1615-8 22825929 PMC3508309

[B27] Bittencourt J. C. (2011). Anatomical organization of the melanin-concentrating hormone peptide family in the mammalian brain. *Gen. Comp. Endocrinol.* 172 185–197 10.1016/j.ygcen.2011.03.028 21463631

[B28] Bittencourt J. C. (2013). The tale of a person and a peptide: Wylie W. Vale Jr. and the role of corticotropin-releasing factor in the stress response. *J. Chem. Neuroanat.* 54 1–4 10.1016/j.jchemneu.2013.04.005 23685258

[B29] Bittencourt J. C. Sawchenko P. E. (2000). Do centrally administered neuropeptides access cognate receptors?: an analysis in the central corticotropin-releasing factor system. *J. Neurosci.* 20 1142–1156 10648719 10.1523/JNEUROSCI.20-03-01142.2000 PMC6774165

[B30] Blasiak A. Blasiak T. Lewandowski M. H. Hossain M. A. Wade J. D. Gundlach A. L. (2013). Relaxin-3 innervation of the intergeniculate leaflet of the rat thalamus – neuronal tract-tracing and in vitro electrophysiological studies. *Eur. J. Neurosci.* 37 1284–1294 10.1111/ejn.12155 23432696

[B31] Blasiak A. Gundlach A. L. Lewandowski M. H. (2010). Orexins increase nucleus incertus neuronal activity: implications for a possible reinforcing arousal drive. *FENS Abstr.* 5 104.2.

[B32] Boels K. Hermans-Borgmeyer I. Schaller H. C. (2004). Identification of a mouse orthologue of the G-protein-coupled receptor SALPR and its expression in adult mouse brain and during development. *Dev. Brain Res.* 152 265–268 10.1016/j.devbrainres.2004.06.002 15351514

[B33] Brundin L. Bjorkqvist M. Petersen A. Traskman-Bendz L. (2007). Reduced orexin levels in the cerebrospinal fluid of suicidal patients with major depressive disorder. *Eur. Neuropsychopharmacol.* 17 573–579 10.1016/j.euroneuro.2007.01.005 17346943

[B34] Burazin T. C. D. Bathgate R. A. D. Macris M. Layfield S. Gundlach A. L. Tregear G. W. (2002). Restricted, but abundant, expression of the novel rat gene-3 (R3) relaxin in the dorsal tegmental region of brain. *J. Neurochem.* 82 1553–1557 10.1046/j.1471-4159.2002.01114.x 12354304

[B35] Burgess C. R. Scammell T. E. (2012). Narcolepsy: neural mechanisms of sleepiness and cataplexy. *J. Neurosci.* 32 12305–12311 10.1523/JNEUROSCI.2630-12.2012 22956821 PMC3449314

[B36] Burt J. Alberto C. O. Parsons M. P. Hirasawa M. (2011). Local network regulation of orexin neurons in the lateral hypothalamus. *Am. J. Physiol. Regul. Integr. Comp. Physiol.* 301 R572–R580 10.1152/ajpregu.00674.2010 21697524

[B37] Caldwell H. K. Lee H. J. Macbeth A. H Young W. S. III (2008). Vasopressin: behavioral roles of an "original" neuropeptide. *Prog. Neurobiol.* 84 1–24 10.1016/j.pneurobio.2007.10.007 18053631 PMC2292122

[B38] Callander G. E. Ma S. Ganella D. E. Wimmer V. C. Gundlach A. L. Thomas W. G. (2012). Silencing relaxin-3 in nucleus incertus of adult rodents: a viral vector-based approach to investigate neuropeptide function. *PLoS ONE* 7:e42300 10.1371/journal.pone.0042300 PMC3410922 22876314

[B39] Carter R. Mouralidarane A. Ray S. Soeda J. Oben J. (2012). Recent advancements in drug treatment of obesity. *Clin. Med.* 12 456–460 10.7861/clinmedicine.12-5-456 23101148 PMC4953770

[B40] Castle D. Keks N. Newton R. Schweitzer I. Copolov D. Paoletti N. (2013). Pharmacological approaches to the management of schizophrenia: 10 years on. *Australas Psychiatry* 21 329–334 10.1177/1039856213486211 23616381

[B41] Cervera-Ferri A. Guerrero-Martinez J. Bataller-Mompean M. Taberner-Cortes A. Martinez-Ricos J. Ruiz-Torner A. (2011). Theta synchronization between the hippocampus and the nucleus incertus in urethane-anesthetized rats. *Exp. Brain Res.* 211 177–192 10.1007/s00221-011-2666-3 21479657

[B42] Cervera-Ferri A. Rahmani Y. Martinez-Bellver S. Teruel-Marti V. Martinez-Ricos J. (2012). Glutamatergic projection from the nucleus incertus to the septohippocampal system. *Neurosci. Lett.* 517 71–76 10.1016/j.neulet.2012.04.014 22521581

[B43] Challis C. Boulden J. Veerakumar A. Espallergues J. Vassoler F. M. Pierce R. C. (2013). Raphe GABAergic neurons mediate the acquisition of avoidance after social defeat. *J. Neurosci.* 33 13978–13988 10.1523/JNEUROSCI.2383-13.2013 23986235 PMC3756748

[B44] Chalmers D. T. Lovenberg T. W De Souza E. B. (1995). Localization of novel corticotropin-releasing factor receptor (CRF2) mRNA expression to specific subcortical nuclei in rat brain: comparison with CRF1 receptor mRNA expression. *J. Neurosci.* 15 6340–6350 7472399 10.1523/JNEUROSCI.15-10-06340.1995 PMC6577987

[B45] Chan L. J. Smith C. M. Chua B. E. Lin F. Bathgate R. A. D. Separovic F. (2013). Synthesis of fluorescent analogues of relaxin family peptides and their preliminary in vitro and in vivo characterization. *Front. Chem.* 1:30 10.3389/fchem.2013.00030 PMC3982560 24790958

[B46] Chee M. J. S. Pissios P. Maratos-Flier E. (2013). Neurochemical characterization of neurons expressing melanin-concentrating hormone receptor 1 in the mouse hypothalamus. *J. Comp. Neurol.* 521 2208–2234 10.1002/cne.23273 23605441 PMC3633152

[B47] Clark L. Chamberlain S. R. Sahakian B. J. (2009). Neurocognitive mechanisms in depression: implications for treatment. *Ann. Rev. Neurosci.* 32 57–74 10.1146/annurev.neuro.31.060407.125618 19400725

[B48] Crawley J. N. Hays S. E. O’Donohue T. L. Paul S. M. Goodwin F. K. (1981). Neuropeptide modulation of social and exploratory behaviors in laboratory rodents. *Peptides *2(Suppl. 1) 123–129 10.1016/0196-9781(81)90066-8 7267400

[B49] Crestani C. C. Alves F. H. F. Gomes F. V. Resstel L. B. M. Correa F. M. A. Herman J. P. (2013). Mechanisms in the bed nucleus of the stria terminalis involved in control of autonomic and neuroendocrine functions: a review. *Curr. Neuropharmacol.* 11 141–159 10.2174/1570159X11311020002 23997750 PMC3637669

[B50] Cummings S. Elde R. Ells J. Lindall A. (1983). Corticotropin-releasing factor immunoreactivity is widely distributed within the central nervous system of the rat: an immunohistochemical study. *J. Neurosci.* 3 1355–1368 6345725 10.1523/JNEUROSCI.03-07-01355.1983 PMC6564441

[B51] Dabrowska J. Hazra R. Guo J.-D. Witt S. D. Rainnie D. G. (2013). Central CRF neurons are not created equal: phenotypic differences in CRF-containing neurons of the rat paraventricular hypothalamus and the bed nucleus of the stria terminalis. *Front. Neurosci. * 7:156 10.3389/fnins.2013.00156 PMC3757458 24009552

[B52] de Wit R. Herrstedt J. Rapoport B. Carides A. D. Guoguang-Ma J. Elmer M. (2004). The oral NK(1) antagonist, aprepitant, given with standard antiemetics provides protection against nausea and vomiting over multiple cycles of cisplatin-based chemotherapy: a combined analysis of two randomised, placebo-controlled phase III clinical trials. *Eur. J. Cancer* 40 403–410 10.1016/S0959-8049(03)00931-6 14746859

[B53] Dimitrov E. L. DeJoseph M. R. Brownfield M. S. Urban J. H. (2007). Involvement of neuropeptide Y Y1 receptors in the regulation of neuroendocrine corticotropin-releasing hormone neuronal activity. *Endocrinology* 148 3666–3673 10.1210/en.2006-1730 17463058

[B54] Domschke K. Reif A. Weber H. Richter J. Hohoff C. Ohrmann P. (2011). Neuropeptide S receptor gene – converging evidence for a role in panic disorder. *Mol. Psychiatry* 16 938–948 10.1038/mp.2010.81 20603625

[B55] Dore R. Iemolo A. Smith K. L. Wang X. Cottone P. Sabino V. (2013). CRF mediates the anxiogenic and anti-rewarding, but not the anorectic effects of PACAP. *Neuropsychopharmacology* 38 2160–2169 10.1038/npp.2013.113 23657440 PMC3773665

[B56] Dunn J. D. (1987). Plasma corticosterone responses to electrical stimulation of the bed nucleus of the stria terminalis. *Brain Res.* 407 327–331 10.1016/0006-8993(87)91111-5 3567648

[B57] Espejo E. F. Minano F. J. (1999). Prefrontocortical dopamine depletion induces antidepressant-like effects in rats and alters the profile of desipramine during Porsolt’s test. *Neuroscience* 88 609–615 10.1016/S0306-4522(98)00258-9 10197779

[B58] Fan J. Bernardi S. Dam N. T. Anagnostou E. Gu X. Martin L. (2012). Functional deficits of the attentional networks in autism. *Brain Behav.* 2 647–660 10.1002/brb3.90 23139910 PMC3489817

[B59] Farooq U. Rajkumar R. Sukumaran S. Wu Y. Tan W. H. Dawe G. S. (2013). Corticotropin-releasing factor infusion into nucleus incertus suppresses medial prefrontal cortical activity and hippocampo-medial prefrontal cortical long-term potentiation. *Eur. J. Neurosci.* 38 2516–2525 10.1111/ejn.12242 23668693

[B60] Farrella M. S. Roth B. L. (2013). Pharmacosynthetics: reimagining the pharmacogenetic approach. *Brain Res.* 1511 6–20 10.1016/j.brainres.2012.09.043 23063887 PMC3562395

[B61] File S. E. Seth P. (2003). A review of 25 years of the social interaction test. *Eur. J. Pharmacol.* 463 35–53 10.1016/S0014-2999(03)01273-1 12600701

[B62] Furutani N. Hondo M. Kageyama H. Tsujino N. Mieda M. Yanagisawa M. (2013). Neurotensin co-expressed in orexin-producing neurons in the lateral hypothalamus plays an important role in regulation of sleep/wakefulness states. *PLoS ONE* 8:e62391 10.1371/journal.pone.0062391 PMC3631195 23620827

[B63] Ganella D. E. Callander G. E. Ma S. Bye C. R. Gundlach A. L Bathgate R. A. D. (2013a). Modulation of feeding by chronic rAAV expression of a relaxin-3 peptide agonist in rat hypothalamus. *Gene Ther.* 20 703–716 10.1038/gt.2012.83 23135160

[B64] Ganella D. E. Ma S. Gundlach A. L. (2013b). Relaxin-3/RXFP3 signaling and neuroendocrine function – a perspective on extrinsic hypothalamic control. *Front. Endocrinol. (Lausanne)* 4:128 10.3389/fendo.2013.00128 PMC3776160 24065955

[B65] Ganella D. E. Ryan P. J. Bathgate R. A. D. Gundlach A. L. (2012). Increased feeding and body weight gain after acute/chronic hypothalamic activation of RXFP3 by relaxin-3 and receptor-selective synthetic and rAAV-driven agonist peptides: functional and therapeutic implications. *Behav. Pharmacol.* 23 516–525 10.1097/FBP.0b013e3283576999 22854307

[B66] Gaus S. E. Strecker R. E. Tate B. A. Parker R. A. Saper C. B. (2002). Ventrolateral preoptic nucleus contains sleep-active, galaninergic neurons in multiple mammalian species. *Neuroscience* 115 285–294 10.1016/S0306-4522(02)00308-1 12401341

[B67] Goto M. Swanson L. W. Canteras N. S. (2001). Connections of the nucleus incertus. *J. Comp. Neurol.* 438 86–122 10.1002/cne.1303 11503154

[B68] Hangya B. Borhegyi Z. Szilágyi N. Freund T. F. Varga V. (2009). GABAergic neurons of the medial septum lead the hippocampal network during theta activity. *J. Neurosci.* 29 8094–8102 10.1523/JNEUROSCI.5665-08.2009 19553449 PMC6666051

[B69] Harrington M. E. (1997). The ventral lateral geniculate nucleus and the intergeniculate leaflet: interrelated structures in the visual and circadian systems. *Neurosci. Biobehav. Rev.* 21 705–727 10.1016/S0149-7634(96)00019-X 9353800

[B70] Hastings M. H. Goedert M. (2013). Circadian clocks and neurodegenerative diseases: time to aggregate? *Curr. Opin. Neurobiol.* 23 1–8 10.1016/j.conb.2013.05.004 23797088 PMC3782660

[B71] Haugaard-Jonsson L. M. Hossain M. A. Daly N. L. Bathgate R. A. D. Wade J. D. Craik D. J. (2008). Structure of the R3/I5 chimeric relaxin peptide, a selective GPBR135 and GPBR142 agonist. *J. Biol. Chem.* 283 23811–23818 10.1074/jbc.M800489200 18577524 PMC3259795

[B72] Haznedar M. M. Buchsbaum M. S. Wei T. C. Hof P. R. Cartwright C. Bienstock C. A. (2000). Limbic circuitry in patients with autism spectrum disorders studied with positron emission tomography and magnetic resonance imaging. *Am. J. Psychiatry* 157 1994–2001 10.1176/appi.ajp.157.12.1994 11097966

[B73] Held K. Antonijevic I. Murck H. Kuenzel H. Steiger A. (2006). Neuropeptide Y (NPY) shortens sleep latency but does not suppress ACTH and cortisol in depressed patients and normal controls. *Psychoneuroendocrinology* 31 100–107 10.1016/j.psyneuen.2005.05.015 16112814

[B74] Hida T. Takahashi E. Shikata K. Hirohashi T. Sawai T. Seiki T. (2006). Chronic intracerebroventricular administration of relaxin-3 increases body weight in rats. *J. Recept. Signal Transduct. Res.* 26 147–158 10.1080/10799890600623373 16777712

[B75] Hisaw F. L. (1926). Experimental relaxation of the pubic ligament of the guinea pig. *Proc. Soc. Exp. Biol. Med.* 23 661–663 10.3181/00379727-23-3107

[B76] Hökfelt T. Bartfai T. Bloom F. (2003). Neuropeptides: opportunities for drug discovery. *Lancet Neurol.* 2 463–472 10.1016/S1474-4422(03)00482-4 12878434

[B77] Hökfelt T. Broberger C. Xu Z.-Q. D. Sergeyev V. Ubink R. Diez M. (2000). Neuropeptides–an overview. *Neuropharmacology* 39 1337–1356 10.1016/S0028-3908(00)00010-1 10818251

[B78] Holmes A. Heilig M. Rupniak N. M. Steckler T. Griebel G. (2003). Neuropeptide systems as novel therapeutic targets for depression and anxiety disorders. *Trends Pharmacol. Sci.* 24 580–588 10.1016/j.tips.2003.09.011 14607081

[B79] Holmes A. J. Lee P. H. Hollinshead M. O. Bakst L. Roffman J. L. Smoller J. W. (2012). Individual differences in amygdala-medial prefrontal anatomy link negative affect, impaired social functioning, and polygenic depression risk. *J. Neurosci.* 32 18087–18100 10.1523/JNEUROSCI.2531-12.2012 23238724 PMC3674506

[B80] Hosken I. T. Smith C. M. Chua B. E. Gundlach A. L. (2013). “Consequences of relaxin-3 null mutation in mice on food-entrainable arousal,” in *Proceedings of Sixth International Conference on Relaxin and Related Peptides* Florence 24640569

[B81] Hossain M. A. Smith C. M. Ryan P. J. Buchler E. Bathgate R. A. D. Gundlach A. L. (2013). Chemical synthesis and orexigenic activity of rat/mouse relaxin-3. *Amino Acids* 44 1529–1536 10.1007/s00726-013-1478-0 23456488

[B82] Hoyer D. Bartfai T. (2012). Neuropeptides and neuropeptide receptors: drug targets, and peptide and non-peptide ligands: a tribute to Prof Dieter Seebach. *Chem. Biodiv.* 9 2367–2387 10.1002/cbdv.201200288 23161624

[B83] Hoyer D. Jacobson L. H. (2013). Orexin in sleep, addiction and more: is the perfect insomnia drug at hand? *Neuropeptides* 47 477–488 10.1016/j.npep.2013.10.009 24215799

[B84] Hryhorczuk C. Sharma S. Fulton S. E. (2013). Metabolic disturbances connecting obesity and depression. *Front. Neurosci.* 7:177 10.3389/fnins.2013.00177 PMC3791387 24109426

[B85] Insel T. Krystal J. Ehlers M. (2013a). New drug development for cognitive enhancement in mental health: challenges and opportunities. *Neuropharmacology* 64 2–7 10.1016/j.neuropharm.2012.07.041 23145450

[B86] Insel T. R. Voon V. Nye J. S. Brown V. J. Altevogt B. M. Bullmore E. T. (2013b). Innovative solutions to novel drug development in mental health. *Neurosci. Biobehav. Rev.* 37 2438–2444 10.1016/j.neubiorev.2013.1003.1022 23563062 PMC3788850

[B87] Ionescu I. A. Dine J. Yen Y. C. Buell D. R. Herrmann L. Holsboer F. (2012). Intranasally administered neuropeptide S (NPS) exerts anxiolytic effects following internalization into NPS receptor-expressing neurons. *Neuropsychopharmacology* 37 1323–1337 10.1038/npp.2011.317 22278093 PMC3327839

[B88] Ishida H. Shirayama Y. Iwata M. Katayama S. Yamamoto A. Kawahara R. (2007). Infusion of neuropeptide Y into CA3 region of hippocampus produces antidepressant-like effect via Y1 receptor. *Hippocampus* 17 271–280 10.1002/hipo.20264 17265460

[B89] Jiang Z. Cowell R. M. Nakazawa K. (2013). Convergence of genetic and environmental factors on parvalbumin-positive interneurons in schizophrenia. *Front. Behav. Neurosci.* 7:116 10.3389/fnbeh.2013.00116 PMC3759852 24027504

[B90] Jones B. E. Halaris A. E. McIlhany M. Moore R. Y. (1977). Ascending projections of the locus coeruleus in the rat. I. Axonal transport in central noradrenaline neurons.* Brain Res.* 127 1–21 10.1016/0006-8993(77)90377-8 67877

[B91] Kalmbach A. Hedrick T. Waters J. (2012). Selective optogenetic stimulation of cholinergic axons in neocortex. *J. Neurophysiol.* 107 2008–2019 10.1152/jn.00870.2011 22236708 PMC3331667

[B92] Keller M. Montgomery S. Ball W. Morrison M. Snavely D. Liu G. (2006). Lack of efficacy of the substance P (neurokinin1 receptor) antagonist Aprepitant in the treatment of major depressive disorder. *Biol. Psychiatry* 59 216–223 10.1016/j.biopsych.2005.07.013 16248986

[B93] Kim A. K. Brown R. M. Lawrence A. J. (2012). The role of orexins/hypocretins in alcohol use and abuse: an appetitive-reward relationship. *Front. Behav. Neurosci*. 6:78 10.3389/fnbeh.2012.00078 PMC3504295 23189046

[B94] Kimura M. Müller-Preuss P. Lu A. Wiesner E. Flachskamm C. Wurst W. (2010). Conditional corticotropin-releasing hormone overexpression in the mouse forebrain enhances rapid eye movement sleep. *Mol. Psychiatry* 15 154–165 10.1038/mp.2009.46 19455148 PMC2834335

[B95] Kirby L. G. Rice K. C. Valentino R. J. (2000). Effects of corticotropin-releasing factor on neuronal activity in the serotonergic dorsal raphe nucleus. *Neuropsychopharmacology* 22 148–162 10.1016/S0893-133X(99)00093-7 10649828

[B96] Kishi T. Elmquist J. K. (2005). Body weight is regulated by the brain: a link between feeding and emotion. *Mol. Psychiatry* 10 132–146 10.1038/sj.mp.4001638 15630408

[B97] Kocsis B. Varga V. Dahan L. Sik A. (2006). Serotonergic neuron diversity: identification of raphe neurons with discharges time-locked to the hippocampal theta rhythm. *Proc. Natl. Acad. Sci. U.S.A.* 103 1059–1064 10.1073/pnas.0508360103 16418294 PMC1347988

[B98] Koob G. F. (2010). The role of CRF and CRF-related peptides in the dark side of addiction. *Brain Res.* 1314 3–14 10.1016/j.brainres.2009.11.008 19912996 PMC2819562

[B99] Kopelman P. G. (2000). Obesity as a medical problem. *Nature* 404 635–643 10766250 10.1038/35007508

[B100] Kuei C. Sutton S. Bonaventure P. Pudiak C. Shelton J. Zhu J. (2007). R3 (BΔ23-27) R/I5 chimeric peptide, a selective antagonist for GPBR135 and GPBR142 over relaxin receptor LGR7. *J. Biol. Chem.* 282 25425 10.1074/jbc.M701416200 17606621

[B101] Lach G de Lima T. C. (2013). Role of NPY Y1 receptor on acquisition, consolidation and extinction on contextual fear conditioning: dissociation between anxiety, locomotion and non-emotional memory behavior. *Neurobiol. Learn. Mem.* 103 26–33 10.1016/j.nlm.2013.04.005 23603424

[B102] Lebow M. Neufeld-Cohen A. Kuperman Y. Tsoory M. Gil S. Chen A. (2012). Susceptibility to PTSD-like behavior is mediated by corticotropin-releasing factor receptor type 2 levels in the bed nucleus of the stria terminalis. *J. Neurosci.* 32 6906–6916 10.1523/JNEUROSCI.4012-11.2012 22593059 PMC6622202

[B103] Lenglos C. Mitra A. Guevremont G. Timofeeva E. (2013). Sex differences in the effects of chronic stress and food restriction on body weight gain and brain expression of CRF and relaxin-3 in rats. *Genes Brain Behav.* 12 370–387 10.1111/gbb.12028 23425370

[B104] Lesch K. P. Waider J. (2012). Serotonin in the modulation of neural plasticity and networks: implications for neurodevelopmental disorders. *Neuron* 76 175–191 10.1016/j.neuron.2012.09.013 23040814

[B105] Li K. Zhou T. Liao L. Yang Z. Wong C. Henn F. (2013). βCaMKII in lateral habenula mediates core symptoms of depression. *Science* 341 1016–1020 10.1126/science.1240729 23990563 PMC3932364

[B106] Lin H. C. Gean P. W. Wang C. C. Chan Y. H. Chen P. S. (2013). The amygdala excitatory/inhibitory balance in a valproate-induced rat autism model. *PLoS ONE* 8:e55248 10.1371/journal.pone.0055248 PMC3558482 23383124

[B107] Lin L. C. Sibille E. (2013). Reduced brain somatostatin in mood disorders: a common pathophysiological substrate and drug target? *Front. Pharmacol.* 4:110 10.3389/fphar.2013.00110 PMC3766825 24058344

[B108] Liu C. Chen J. Kuei C. Sutton S. Nepomuceno D. Bonaventure P. (2005). Relaxin-3/insulin-like peptide 5 chimeric peptide, a selective ligand for G protein-coupled receptor (GPCR)135 and GPBR142 over leucine-rich repeat-containing G protein-coupled receptor 7. *Mol. Pharmacol.* 67 231–240 10.1124/mol.104.006700 15465925

[B109] Liu C. Eriste E. Sutton S. Chen J. Roland B. Kuei C. (2003). Identification of relaxin-3/INSL7 as an endogenous ligand for the orphan G-protein-coupled receptor GPBR135. *J. Biol. Chem.* 278 50754–50764 10.1074/jbc.M308995200 14522968

[B110] Liu C. Kuei C. Sutton S. Shelton J. Zhu J. Nepomuceno D. (2009). Probing the functional domains of relaxin-3 and the creation of a selective antagonist for RXFP3/GPBR135 over relaxin receptor RXFP1/LGR7. *Ann. N. Y. Acad. Sci.* 1160 31–37 10.1111/j.1749-6632.2008.03790.x 19416155

[B111] Liu T. Wang Q. Berglund E. D. Tong Q. (2013). Action of neurotransmitter: a key to unlock the AgRP neuron feeding circuit. *Front. Neurosci.* 6:200 10.3389/fnins.2012.00200 PMC3549528 23346045

[B112] Lukas M. Neumann I. D. (2013). Oxytocin and vasopressin in rodent behaviors related to social dysfunctions in autism spectrum disorders. *Behav. Brain Res.* 251 85–94 10.1016/j.bbr.2012.08.011 22981649

[B113] Lukas M. Toth I. Reber S. O. Slattery D. A. Veenema A. H. Neumann I. D. (2011). The neuropeptide oxytocin facilitates pro-social behavior and prevents social avoidance in rats and mice. *Neuropsychopharmacology* 36 2159–2168 10.1038/npp.2011.95 21677650 PMC3176581

[B114] Lungwitz E. A. Molosh A. Johnson P. L. Harvey B. P. Dirks R. C. Dietrich A. (2012). Orexin-A induces anxiety-like behavior through interactions with glutamatergic receptors in the bed nucleus of the stria terminalis of rats. *Physiol. Behav.* 107 726–732 10.1016/j.physbeh.2012.05.019 22652097 PMC3482273

[B115] Ma S. Blasiak A. Olucha-Bordonau F. E. Verberne A. J. Gundlach A. L. (2013). Heterogeneous responses of nucleus incertus neurons to corticotropin-releasing factor and coherent activity with hippocampal theta rhythm in the rat. *J. Physiol. (Lond.)* 591 3981–4001 10.1113/jphysiol.2013.254300 23671163 PMC3764641

[B116] Ma S. Bonaventure P. Ferraro T. Shen P. J. Burazin T. C. D. Bathgate R. A. D. (2007). Relaxin-3 in GABA projection neurons of nucleus incertus suggests widespread influence on forebrain circuits via G-protein-coupled receptor-135 in the rat. *Neuroscience* 144 165–190 10.1016/j.neuroscience.2006.08.072 17071007

[B117] Ma S. Kastman H. Olucha-Bordonau F. E. Capogna M. Hossain A. Wade J. D. (2010). Relaxin-3 receptor activation in the central amygdala enhances fear extinction in the rat: implications for relaxin-3 control of emotion. *Soc. Neurosci. Abstr.* 809.24

[B118] Ma S. Olucha-Bordonau F. E. Hossain M. A. Lin F. Kuei C. Liu C. (2009a). Modulation of hippocampal theta oscillations and spatial memory by relaxin-3 neurons of the nucleus incertus. *Learn. Mem.* 16 730–742 10.1101/lm.1438109 19880588

[B119] Ma S. Sang Q. Lanciego J. L. Gundlach A. L. (2009b). Localization of relaxin-3 in brain of Macaca fascicularis - Identification of nucleus incertus in primate. *J. Comp. Neurol.* 517 700–712 10.1002/cne.22197 19844992

[B120] Ma S. Shen P. J. Sang Q. Lanciego J. L. Gundlach A. L. (2009c). Distribution of relaxin-3 mRNA and immunoreactivity and RXFP3-binding sites in the brain of the macaque, Macaca fascicularis. *Ann. N. Y. Acad. Sci.* 1160 256–258 10.1111/j.1749-6632.2009.03954.x 19416198

[B121] Maaswinkel H. Gispen W. H. Spruijt B. M. (1997). Executive function of the hippocampus in social behavior in the rat. *Behav. Neurosci.* 111 777–784 10.1037/0735-7044.111.4.777 9267654

[B122] Manji H. K. Quiroz J. A. Sporn J. Payne J. L. Denicoff K. Gray A. N. (2003). Enhancing neuronal plasticity and cellular resilience to develop novel, improved therapeutics for difficult-to-treat depression. *Biol. Psychiatry* 53 707–742 10.1016/S0006-3223(03)00117-3 12706957

[B123] Marchant E. G. Watson N. V. Mistlberger R. E. (1997). Both neuropeptide Y and serotonin are necessary for entrainment of circadian rhythms in mice by daily treadmill running schedules. *J. Neurosci.* 17 7974 –7987 9315915 10.1523/JNEUROSCI.17-20-07974.1997 PMC6793923

[B124] Marder E. (2012). Neuromodulation of neuronal circuits: back to the future. *Neuron* 76 1–11 10.1016/j.neuron.2012.09.010 23040802 PMC3482119

[B125] Markram K. Markram H. (2010). The intense world theory – a unifying theory of the neurobiology of autism. *Front. Hum. Neurosci.* 4:224 10.3389/fnhum.2010.00224 PMC3010743 21191475

[B126] Marson L. Foley K. A. (2004). Identification of neural pathways involved in genital reflexes in the female: a combined anterograde and retrograde tracing study. *Neuroscience* 127 723–736 10.1016/j.neuroscience.2004.04.063 15283970

[B127] Matsumoto M. Kamohara M. Sugimoto T. Hidaka K. Takasaki J. Saito T. (2000). The novel G-protein coupled receptor SALPR shares sequence similarity with somatostatin and angiotensin receptors. *Gene* 248 183–189 10.1016/S0378-1119(00)00123-2 10806363

[B128] Mazure C. M. (1998). Life stressors as risk factors in depression. *Clin. Psychol. Sci. Pract.* 5 291–313 10.1111/j.1468-2850.1998.tb00151.x

[B129] McClung C. A. (2007). Circadian genes, rhythms and the biology of mood disorders. *Pharmacol. Ther.* 114 222–232 10.1016/j.pharmthera.2007.02.003 17395264 PMC1925042

[B130] McEwen B. S. (2007). Physiology and neurobiology of stress and adaptation: central role of the brain. *Physiol. Rev.* 87 873–904 10.1152/physrev.00041.2006 17615391

[B131] McGonigle P. (2011). Peptide therapeutics for CNS indications. *Biochem. Pharmacol.* 83 559–566 10.1016/j.bcp.2011.10.014 22051078

[B132] McGowan B. M. Stanley S. A. Smith K. L. Minnion J. S. Donovan J. Thompson E. L. (2006). Effects of acute and chronic relaxin-3 on food intake and energy expenditure in rats. *Regul. Peptides* 136 72–77 10.1016/j.regpep.2006.04.009 16764952

[B133] McGowan B. M. Stanley S. A. Smith K. L. White N. E. Connolly M. M. Thompson E. L. (2005). Central relaxin-3 administration causes hyperphagia in male Wistar rats. *Endocrinology* 146 3295–3300 10.1210/en.2004-1532 15845619

[B134] McGowan B. M. Stanley S. A. White N. E. Spangeus A. Patterson M. Thompson E. L. (2007). Hypothalamic mapping of orexigenic action and Fos-like immunoreactivity following relaxin-3 administration in male Wistar rats. *Am. J. Physiol. Endocrinol. Metab.* 292 E913–E919 10.1152/ajpendo.00346.2006 17132825

[B135] McNamara I. M. Borella A. W. Bialowas L. A. Whitaker-Azmitia P. M. (2008). Further studies in the developmental hyperserotonemia model (DHS) of autism: social, behavioral and peptide changes. *Brain Res.* 1189 203–214 10.1016/j.brainres.2007.10.063 18062943

[B136] McNaughton N. Gray J. A. (2000). Anxiolytic action on the behavioural inhibition system implies multiple types of arousal contribute to anxiety. *J. Affect. Disord.* 61 161–176 10.1016/S0165-0327(00)00344-X 11163419

[B137] McNaughton N. Kocsis B. Hajos M. (2007). Elicited hippocampal theta rhythm: a screen for anxiolytic and procognitive drugs through changes in hippocampal function? *Behav. Pharmacol.* 18 329–346 10.1097/FBP.0b013e3282ee82e3 17762505

[B138] Meyer-Bernstein E. L. Morin L. P. (1996). Differential serotonergic innervation of the suprachiasmatic nucleus and the intergeniculate leaflet and its role in circadian rhythm modulation. *J. Neurosci.* 16 2097–2111 8604054 10.1523/JNEUROSCI.16-06-02097.1996 PMC6578502

[B139] Millan M. J. Agid Y. Brüne M. Bullmore E. T. Carter C. S. Clayton N. S. (2012). Cognitive dysfunction in psychiatric disorders: characteristics, causes and the quest for improved therapy. *Nat. Rev. Drug Discov.* 11 141–168 10.1038/nrd3628 22293568

[B140] Miyamoto Y. Watanabe Y. Tanaka M. (2008). Developmental expression and serotonergic regulation of relaxin 3/INSL7 in the nucleus incertus of rat brain. *Regul. Peptides* 145 54–59 10.1016/j.regpep.2007.08.010 17870193

[B141] Mogi K. Nagasawa M. Kikusui T. (2010). Developmental consequences and biological significance of mother-infant bonding. *Prog. Neuropsychopharmacol. Biol. Psychiatry* 35 1232–1241 10.1016/j.pnpbp.2010.08.024 20817069

[B142] Möller C. Sommer W. Thorsell A. Heilig M. (1999). Anxiogenic-like action of galanin after intra-amygdala administration in the rat. *Neuropsychopharmacology* 21 507–512 10.1016/S0893-133X(98)00102-X 10481834

[B143] Monti J. M. Jantos H. (2008). The roles of dopamine and serotonin, and of their receptors, in regulating sleep and waking. *Prog. Brain Res.* 172 625–646 10.1016/S0079-6123(08)00929-1 18772053

[B144] Monti J. M. Monti D. (2000). Sleep disturbance in generalized anxiety disorder and its treatment. *Sleep Med. Rev.* 4 263–276 10.1053/smrv.1999.0096 12531169

[B145] Morin L. P. (2013). Neuroanatomy of the extended circadian rhythm system. *Exp. Neurol.* 243 4–20 10.1016/j.expneurol.2012.06.026 22766204 PMC3498572

[B146] Morin L. P. Meyer-Bernstein E. L. (1999). The ascending serotonergic system in the hamster: comparison with projections of the dorsal and median raphe nuclei. *Neuroscience* 91 81–105 10.1016/S0306-4522(98)00585-5 10336062

[B147] Morin S. M. Ling N. Liu X. J. Kahl S. D. Gehlert D. R. (1999). Differential distribution of urocortin- and corticotropin-releasing factor-like immunoreactivities in the rat brain. *Neuroscience* 92 281–291 10.1016/S0306-4522(98)00732-5 10392850

[B148] Munro J. Skrobot O. Sanyoura M. Kay V. Susce M. T. Glaser P. E. (2012). Relaxin polymorphisms associated with metabolic disturbance in patients treated with antipsychotics. *J. Psychopharmacol.* 26 374–379 10.1177/0269881111408965 21693553

[B149] Murphy F. C. Rubinsztein J. S. Michael A. Rogers R. D. Robbins T. W. Paykel E. S. (2001). Decision-making cognition in mania and depression. *Psychol. Med.* 31 679–693 10.1017/S0033291701003804 11352370

[B150] Nakazawa C. M. Shikata K. Uesugi M. Katayama H. Aoshima K. Tahara K. (2013). Prediction of relaxin-3-induced downstream pathway resulting in anxiolytic-like behaviors in rats based on a microarray and peptidome analysis. *J. Recept. Signal Transduct. Res.* 33 224–233 10.3109/10799893.2012.756895 23697547

[B151] Nawaratne V. Leach K. Suratman N. Loiacono R. E. Felder C. C. Armbruster B. N. (2008). New insights into the function of M4 muscarinic acetylcholine receptors gained using a novel allosteric modulator and a DREADD (designer receptor exclusively activated by a designer drug). *Mol. Pharmacol.* 74 1119–1131 10.1124/mol.108.049353 18628403

[B152] Nemeroff C. B. (1992). New vistas in neuropeptide research in neuropsychiatry: focus on corticotropin-releasing factor. *Neuropsychopharmacology* 6 69–75 1610487

[B153] Nestler E. J. (1998). Antidepressant treatments in the 21st century. *Biol. Psychiatry* 44 526–533 10.1016/S0006-3223(98)00095-X 9787876

[B154] Nestler E. J. Hyman S. E. (2010). Animal models of neuropsychiatric disorders. *Nat. Neurosci.* 13 1161–1169 10.1038/nn.2647 20877280 PMC3750731

[B155] Nikisch G. Baumann P. Liu T. Mathe A. A. (2011). Quetiapine affects neuropeptide Y and corticotropin-releasing hormone in cerebrospinal fluid from schizophrenia patients: relationship to depression and anxiety symptoms and to treatment response. *Int. J. Neuropsychopharmacol.* 15 1051–1061 10.1017/S1461145711001556 22008251

[B156] Nunez A. Cervera-Ferri A. Olucha-Bordonau F. Ruiz-Torner A. Teruel V. (2006). Nucleus incertus contribution to hippocampal theta rhythm generation. *Eur. J. Neurosci.* 23 2731–2738 10.1111/j.1460-9568.2006.04797.x 16817876

[B157] Nutt D. J. Forshall S. Bell C. Rich A. Sandford J. Nash J. (1999). Mechanisms of action of selective serotonin reuptake inhibitors in the treatment of psychiatric disorders. *Eur. Neuropsychopharmacol.* 9 S81–S86 10.1016/S0924-977X(99)00030-9 10523062

[B158] O’Donnell P. (2011). Adolescent onset of cortical disinhibition in schizophrenia: insights from animal models. *Schizophr. Bull.* 37 484–492 10.1093/schbul/sbr028 21505115 PMC3080677

[B159] Ohnishi T. Matsuda H. Hashimoto T. Kunihiro T. Nishikawa M. Uema T. (2000). Abnormal regional cerebral blood flow in childhood autism. *Brain* 123 1838–1844 10.1093/brain/123.9.1838 10960047

[B160] Olucha-Bordonau F. E. Otero-Garcia M. Sanchez-Perez A. M. Nunez A. Ma S. Gundlach A. L. (2012). Distribution and targets of the relaxin-3 innervation of the septal area in the rat. *J. Comp. Neurol.* 520 1903–1939 10.1002/cne.23018 22134882

[B161] Olucha-Bordonau F. E. Teruel V. Barcia-Gonzalez J. Ruiz-Torner A. Valverde-Navarro A. A. Martinez-Soriano F. (2003). Cytoarchitecture and efferent projections of the nucleus incertus of the rat. *J. Comp. Neurol.* 464 62–97 10.1002/cne.10774 12866129

[B162] Paez-Pereda M. Hausch F. Holsboer F. (2011). Corticotropin releasing factor receptor antagonists for major depressive disorder. *Expert Opin. Investig. Drugs* 20 519–535 10.1517/13543784.2011.565330 21395482

[B163] Panchal S. K. Brown L. (2011). Rodent models for metabolic syndrome research. *J. Biomed. Biotechnol.* 35 1982 10.1155/2011/351982 PMC3018657 21253582

[B164] Pape H.-C. Jüngling K. Seidenbecher T. Lesting J. Reinscheid R. K. (2010). Neuropeptide S: a transmitter system in the brain regulating fear and anxiety. *Neuropharmacology* 58 29–34 10.1016/j.neuropharm.2009.06.001 19523478 PMC2784192

[B165] Peca J. Feliciano C. Ting J. T. Wang W. Wells M. F. Venkatraman T. N. (2011). Shank3 mutant mice display autistic-like behaviours and striatal dysfunction. *Nature* 472 437–442 10.1038/nature09965 21423165 PMC3090611

[B166] Pekala D. Blasiak T. Raastad M. Lewandowski M. H. (2011). The influence of orexins on the firing rate and pattern of rat intergeniculate leaflet neurons - electrophysiological and immunohistological studies. *Eur. J. Neurosci.* 34 1406–1418 10.1111/j.1460-9568.2011.07868.x 22034975

[B167] Pereira C. W. Santos F. N. Sanchez-Perez A. M. Otero-Garcia M. Marchioro M. Ma S. (2013). Electrolytic lesion of the nucleus incertus retards extinction of auditory conditioned fear. *Behav. Brain Res.* 247 201–210 10.1016/j.bbr.2013.03.025 23538065

[B168] Peyron C. Tighe D. K. van den Pol A. N. de Lecea L. Heller H. C. Sutcliffe J. G. (1998). Neurons containing hypocretin (orexin) project to multiple neuronal systems. *J. Neurosci.* 18 9996–10015 9822755 10.1523/JNEUROSCI.18-23-09996.1998 PMC6793310

[B169] Pulga A. Ruzza C. Rizzi A. Guerrini R. Calo G. (2012). Anxiolytic- and panicolytic-like effects of neuropeptide S in the mouse elevated T-maze. *Eur. J. Neurosci.* 36 3531–3537 10.1111/j.1460-9568.2012.08265.x 22928868

[B170] Rajkumar R. See L. K. Dawe G. S. (2013). Acute antipsychotic treatments induce distinct c-Fos expression patterns in appetite-related neuronal structures of the rat brain. *Brain Res.* 1508 34–43 10.1016/j.brainres.2013.02.050 23499563

[B171] Ressler K. J. Mercer K. B. Bradley B. Jovanovic T. Mahan A. Kerley K. (2011). Post-traumatic stress disorder is associated with PACAP and the PAC1 receptor. *Nature* 470 492–497 10.1038/nature09856 21350482 PMC3046811

[B172] Ring R. H. (2011). A complicated picture of oxytocin action in the central nervous system revealed. *Biol. Psychiatry* 69 818–819 10.1016/j.biopsych.2011.03.020 21497680

[B173] Rosengren K. J. Lin F. Bathgate R. A. D. Tregear G. W. Daly N. L. Wade J. D. (2006). Solution structure and novel insights into the determinants of the receptor specificity of human relaxin-3. *J. Biol. Chem.* 281 5845–5851 10.1074/jbc.M511210200 16365033

[B174] Rotzinger S. Lovejoy D. A. Tan L. A. (2010). Behavioral effects of neuropeptides in rodent models of depression and anxiety. *Peptides* 31 736–756 10.1016/j.peptides.2009.12.015 20026211

[B175] Roux L. Donaldson C. (2012). Economics and obesity: costing the problem or evaluating solutions? *Obes. Res.* 12 173–179 10.1038/oby.2004.23 14981208

[B176] Russo S. J. Nestler E. J. (2013). The brain reward circuitry in mood disorders. *Nat. Rev. Neurosci.* 14 609–625 10.1038/nrn3381 23942470 PMC3867253

[B177] Ryan P. J. Buchler E. Shabanpoor F. Hossain M. A. Wade J. D. Lawrence A. J. (2013a). Central relaxin-3 receptor (RXFP3) activation decreases anxiety- and depressive-like behaviours in the rat. *Behav. Brain Res.* 244 142–151 10.1016/j.bbr.2013.01.034 23380674

[B178] Ryan P. J. Kastman H. E. Krstrew E. V. Chirlov L. Rosengren K. J. Hossain M. A. (2013b). Relaxin-3/RXFP3 system regulates alcohol-seeking. *Proc. Natl. Acad. Sci. U.S.A.* 110 20789–20794 10.1073/pnas.1317807110 24297931 PMC3870696

[B179] Ryan P. J. Ma S. Olucha-Bordonau F. E. Gundlach A. L. (2011). Nucleus incertus – an emerging modulatory role in arousal, stress and memory. *Neurosci. Biobehav. Rev.* 35 1326–1341 10.1016/j.neubiorev.2011.02.004 21329721

[B180] Saito Y. Nagasaki H. (2008). The melanin-concentrating hormone system and its physiological functions. *Results Probl. Cell Differ.* 46 159–179 10.1007/400_2007_052 18227983

[B181] Sakurai T. (2007). The neural circuit of orexin (hypocretin): maintaining sleep and wakefulness. *Nat. Rev. Neurosci.* 8 171–181 10.1038/nrn2092 17299454

[B182] Sanogo Y. O. Hankison S. Band M. Obregon A. Bell A. M. (2011). Brain transcriptomic response of threespine sticklebacks to cues of a predator. *Brain Behav. Evol.* 77 270–285 10.1159/000328221 21677424 PMC3182040

[B183] Saper C. B. Scammell T. E. Lu J. (2005). Hypothalamic regulation of sleep and circadian rhythms. *Nature* 437 1257–1263 10.1038/nature04284 16251950

[B184] Sasaki K. Suzuki M. Mieda M. Tsujino N. Roth B. Sakurai T. (2011). Pharmacogenetic modulation of orexin neurons alters sleep/wakefulness states in mice. *PLoS ONE* 6:e20360 10.1371/journal.pone.0020360 PMC3103553 21647372

[B185] Schmidt H. D. Duman R. S. (2010). Peripheral BDNF produces antidepressant-like effects in cellular and behavioral models. *Neuropsychopharmacology* 35 2378–2391 10.1038/npp.2010.114 20686454 PMC2955759

[B186] Shabanpoor F. Akhter Hossain M. Ryan P. J. Belgi A. Layfield S. Kocan M. (2012). Minimization of human relaxin-3 leading to high-affinity analogues with increased selectivity for relaxin-family peptide 3 receptor (RXFP3) over RXFP1. *J. Med. Chem.* 55 1671–1681 10.1021/jm201505p 22257012

[B187] Sherwood O. D. (2004). Relaxin’s physiological roles and other diverse actions. *Endocr. Rev.* 25 205–234 10.1210/er.2003-0013 15082520

[B188] Shinohara K. Tominaga K. Isobe Y lnouye S.-I. T. (1993). Photic regulation of peptides located in the ventrolateral subdivision of the suprachiasmatic nucleus of the rat: daily variations of vasoactive intestinal polypeptide, gastrin-releasing peptide, and neuropeptide Y. *J. Neurosci.* 13 793–800 8426236 10.1523/JNEUROSCI.13-02-00793.1993 PMC6576648

[B189] Silverman J. L. Tolu S. S. Barkan C. L. Crawley J. N. (2010). Repetitive self-grooming behavior in the BTBR mouse model of autism is blocked by the mGluR5 antagonist MPEP. *Neuropsychopharmacology* 35 976–989 10.1038/npp.2009.201 20032969 PMC2827881

[B190] Smith C. M. Blasiak A. Ganella D. E. Chua B. E. Layfield S. L. Bathgate R. A. D. (2013a). “Viral-mediated delivery of an RXFP3 agonist into brain promotes arousal in mice,” in *Proceedings of Sixth International Conference on Relaxin and Related Peptides* Florence 24640570

[B191] Smith C. M. Chua B. E. Walker A. W. Gundlach A. L. (2013b). “Potential hypothalamic targets of relaxin-3 innervation: a perspective,” in *Proceedings of Sixth International Conference on Relaxin and Related Peptides* Florence 24640571

[B192] Smith C. M. Hosken I. T. Sutton S. W. Lawrence A. J. Gundlach A. L. (2012). Relaxin-3 null mutation mice display a circadian hypoactivity phenotype. *Genes Brain Behav.* 11 94–104 10.1111/j.1601-183X.2011.00730.x 21899720

[B193] Smith C. M. Lawrence A. J. Sutton S. W. Gundlach A. L. (2009). Behavioral phenotyping of mixed-background (129S5:B6) relaxin-3 knockout mice. *Ann. N. Y. Acad. Sci.* 1160 236–241 10.1111/j.1749-6632.2009.03953.x 19416195

[B194] Smith C. M. Ryan P. J. Hosken I. T. Ma S. Gundlach A. L. (2011). Relaxin-3 systems in the brain – the first 10 years. *J. Chem. Neuroanat.* 42 262–275 10.1016/j.jchemneu.2011.05.013 21693186

[B195] Smith C. M. Shen P. J. Banerjee A. Bonaventure P. Ma S. Bathgate R. A. D. (2010). Distribution of relaxin 3 and RXFP3 within arousal, stress, affective, and cognitive circuits of mouse brain. *J. Comp. Neurol.* 518 4016–4045 10.1002/cne.22442 20737598

[B196] Smith K. L. Patterson M. Dhillo W. S. Patel S. R. Semjonous N. M. Gardiner J. V. (2006). Neuropeptide S stimulates the hypothalamo-pituitary-adrenal axis and inhibits food intake. *Endocrinology* 147 3510–3518 10.1210/en.2005-1280 16574794

[B197] Sodersten P. Bergh C. Zandian M. (2006). Understanding eating disorders. *Horm. Behav.* 50 572–578 10.1016/j.yhbeh.2006.06.030 16890228

[B198] Spencer K. M. Nestor P. G. Niznikiewicz M. A. Salisbury D. F. Shenton M. E. McCarley R. W. (2003). Abnormal neural synchrony in schizophrenia. *J. Neurosci.* 23 7407–7411 12917376 10.1523/JNEUROSCI.23-19-07407.2003 PMC2848257

[B199] Steinbusch H. W. (1981). Distribution of serotonin-immunoreactivity in the central nervous system of the rat-cell bodies and terminals. *Neuroscience* 6 557–618 10.1016/0306-4522(81)90146-9 7017455

[B200] Sutton S. W. Bonaventure P. Kuei C. Roland B. Chen J. Nepomuceno D. (2004). Distribution of G-protein-coupled receptor (GPCR)135 binding sites and receptor mRNA in the rat brain suggests a role for relaxin-3 in neuroendocrine and sensory processing. *Neuroendocrinology* 80 298–307 10.1159/000083656 15677880

[B201] Sutton S. W. Shelton J. Smith C. Williams J. Yun S. Motley T. (2009). Metabolic and neuroendocrine responses to RXFP3 modulation in the central nervous system. *Ann. N. Y. Acad. Sci.* 1160 242–249 10.1111/j.1749-6632.2008.03812.x 19416196

[B202] Takagi H. Shiosaka S. Tohyama M. Senba E. Sakanaka M. (1980). Ascending components of the medial forebrain bundle from the lower brain stem in the rat, with special reference to raphe and catecholamine cell groups. A study by the HRP method.* Brain Res.* 193 315–337 10.1016/0006-8993(80)90168-7 6966962

[B203] Tanaka M. (2010). Relaxin-3/insulin-like peptide 7, a neuropeptide involved in the stress response and food intake. *FEBS J.* 277 4990–4997 10.1111/j.1742-4658.2010.07931.x 21126312

[B204] Tanaka M. Iijima N. Miyamoto Y. Fukusumi S. Itoh Y. Ozawa H. (2005). Neurons expressing relaxin 3/INSL 7 in the nucleus incertus respond to stress. *Eur. J. Neurosci.* 21 1659–1670 10.1111/j.1460-9568.2005.03980.x 15845093

[B205] Tanaka M. Watanabe Y. Yoshimoto K. (2009). Regulation of relaxin 3 gene expression via cAMP-PKA in a neuroblastoma cell line. *J. Neurosci. Res.* 87 820–829 10.1002/jnr.21895 18831067

[B206] Tandon R. (2011). Antipsychotics in the treatment of schizophrenia: an overview. *J. Clin. Psychiatry *72(Suppl. 1) 4–8 10.4088/JCP.10075su1.01 22217436

[B207] Teruel-Marti V. Cervera-Ferri A. Nunez A. Valverde-Navarro A. A. Olucha-Bordonau F. E. Ruiz-Torner A. (2008). Anatomical evidence for a ponto-septal pathway via the nucleus incertus in the rat. *Brain Res.* 1218 87–96 10.1016/j.brainres.2008.04.022 18514169

[B208] Thankachan S. Rusak B. (2005). Juxtacellular recording/labeling analysis of physiological and anatomical characteristics of rat intergeniculate leaflet neurons. *J. Neurosci.* 25 9195–9204 10.1523/JNEUROSCI.2672-05.2005 16207879 PMC6725760

[B209] Theisen F. M. Linden A. Konig I. R. Martin M. Remschmidt H. Hebebrand J. (2003). Spectrum of binge eating symptomatology in patients treated with clozapine and olanzapine. *J. Neural Transm.* 110 111–121 12541016 10.1007/s00702-002-0792-6

[B210] Vaccari C. Lolait S. J. Ostrowski N. L. (1998). Comparative distribution of vasopressin V1b and oxytocin receptor messenger ribonucleic acids in brain. *Endocrinology* 139 5015–5033 9832441 10.1210/endo.139.12.6382

[B211] Van Cauter E. Linkowski P. Kerkhofs M. Hubain P. L’Hermite-Baleriaux M. Leclercq R. (1991). Circadian and sleep-related endocrine rhythms in schizophrenia. *Arch. Gen. Psychiatry* 48 348 10.1001/archpsyc.1991.01810280064009 1848971

[B212] van den Pol A. N. (2012). Neuropeptide transmission in brain circuits. *Neuron* 76 98–115 10.1016/j.neuron.2012.09.014 23040809 PMC3918222

[B213] van der Westhuizen E. T. Christopoulos A. Sexton P. M. Wade J. D. Summers R. J. (2010). H2 relaxin is a biased ligand relative to H3 relaxin at the relaxin family peptide receptor 3 (RXFP3). *Mol. Pharmacol.* 77 759–772 10.1124/mol.109.061432 20159943

[B214] van der Westhuizen E. T. Werry T. D. Sexton P. M. Summers R. J. (2007). The relaxin family peptide receptor 3 activates extracellular signal-regulated kinase 1/2 through a protein kinase C-dependent mechanism. *Mol. Pharmacol.* 71 1618–1629 10.1124/mol.106.032763 17351017

[B215] van Elst L. T. Woermann F. G. Lemieux L. Thompson P. J. Trimble M. R. (2000). Affective aggression in patients with temporal lobe epilepsy: a quantitative MRI study of the amygdala. *Brain* 123 234–243 10.1093/brain/123.2.234 10648432

[B216] Van Pett K. Viau V. Bittencourt J. C. Chan R. K. Li H. Y. Arias C. (2000). Distribution of mRNAs encoding CRF receptors in brain and pituitary of rat and mouse. *J. Comp. Neurol.* 428 191–212 10.1002/1096-9861(20001211)428:2<191::AID-CNE1>3.0.CO;2-U 11064361

[B217] Vertes R. P. Kocsis B. (1997). Brainstem-diencephalo-septohippocampal systems controlling the theta rhythm of the hippocampus. *Neuroscience* 81 893–926 9330355 10.1016/s0306-4522(97)00239-x

[B218] Vianna D. M. Graeff F. G. Landeira-Fernandez J. Brandao M. L. (2001). Lesion of the ventral periaqueductal gray reduces conditioned fear but does not change freezing induced by stimulation of the dorsal periaqueductal gray. *Learn. Mem.* 8 164–169 10.1101/lm.36101 11390636 PMC311373

[B219] Videbech P. Ravnkilde B. (2004). Hippocampal volume and depression: a meta-analysis of MRI studies. *Am. J. Psychiatry* 161 1957–1966 10.1176/appi.ajp.161.11.1957 15514393

[B220] Vithlani M. Hines R. M. Zhong P. Terunuma M. Hines D. J. Revilla-Sanchez R. (2013). The ability of BDNF to modify neurogenesis and depressive-like behaviors is dependent upon phosphorylation of tyrosine residues 365/367 in the GABAA-receptor γ2 subunit. *J. Neurosci.* 33 15567–15577 10.1523/JNEUROSCI.1845-13.2013 24068823 PMC3782626

[B221] von Euler U. S. Gaddum J. H. (1931). An unidentified depressor substance in certain tissue extracts. *J. Physiol.* 72 74 10.1113/jphysiol.1931.sp002763 PMC1403098 16994201

[B222] Walker D. L. Miles L. A. Davis M. (2009). Selective participation of the bed nucleus of the stria terminalis and CRF in sustained anxiety-like vs. phasic fear-like responses. *Prog. Neuropsychopharmacol. Biol. Psychiatry* 33 1291–1308 10.1016/j.pnpbp.2009.06.022 PMC2783512 19595731

[B223] Wang X. J. (2002). Pacemaker neurons for the theta rhythm and their synchronization in the septohippocampal reciprocal loop. *J. Neurophysiol.* 87 889–900 11826054 10.1152/jn.00135.2001

[B224] Watanabe Y. Miyamoto Y. Matsuda T. Tanaka M. (2011a). Relaxin-3/INSL7 regulates the stress-response system in the rat hypothalamus. *J. Mol. Neurosci.* 43 169–174 10.1007/s12031-010-9468-0 21072619

[B225] Watanabe Y. Tsujimura A. Takao K. Nishi K. Ito Y. Yasuhara Y. (2011b). Relaxin-3-deficient mice showed slight alteration in anxiety-related behavior. *Front. Behav. Neurosci.* 5:50 10.3389/fnbeh.2011.00050 PMC3156976 21887138

[B226] Wess J. Nakajima K. Jain S. (2013). Novel designer receptors to probe GPCR signaling and physiology. *Trends Pharmacol. Sci.* 34 385–392 10.1016/j.tips.2013.04.006 23769625 PMC3758874

[B227] Westenberg H. G. (1999). Pharmacology of antidepressants: selectivity or multiplicity? *J. Clin. Psychiatry *60(Suppl. 17) 4–8 10446734

[B228] Wilkinson T. N. Speed T. P. Tregear G. W Bathgate R. A. D. (2005). Evolution of the relaxin-like peptide family. *BMC Evol. Biol.* 5:14 10.1186/1471-2148-5-14 PMC551602 15707501

[B229] Willard S. L. Shively C. A. (2012). Modeling depression in adult female cynomolgus monkeys (Macaca fascicularis). *Am. J. Primatol.* 74 528–542 10.1002/ajp.21013 22076882

[B230] Willner P. Scheel-Krüger J. Belzung C. (2013). The neurobiology of depression and antidepressant action. *Neurosci. Biobehav. Rev.* 37 2331–2371 10.1016/j.neubiorev.2012.12.007 23261405

[B231] Winrow C. J. Renger J. J. (2014). Discovery and development of orexin receptor antagonists as therapeutics for insomnia. *Br. J. Pharmacol.* 171 283–293 10.1111/bph.12261 23731216 PMC3904252

[B232] Xu Y. L. Reinscheid R. K. Huitron-Resendiz S. Clark S. D. Wang Z. Lin S. H. (2004). Neuropeptide S: a neuropeptide promoting arousal and anxiolytic-like effects. *Neuron* 43 487–497 10.1016/j.neuron.2004.08.005 15312648

[B233] Yamasue H. Yee J. R. Hurlemann R. Rilling J. K. Chen F. S. Meyer-Lindenberg A. (2012). Integrative approaches utilizing oxytocin to enhance prosocial behavior: from animal and human social behavior to autistic social dysfunction. *J. Neurosci.* 32 14109–14117 10.1523/JNEUROSCI.3327-12.2012 23055480 PMC6622380

[B234] Yizhar O. Fenno L. E. Davidson T. J. Mogri M. Deisseroth K. (2011a). Optogenetics in neural systems. *Neuron* 71 9–34 10.1016/j.neuron.2011.06.004 21745635

[B235] Yizhar O. Fenno L. E. Prigge M. Schneider F. Davidson T. J. O’Shea D. J. (2011b). Neocortical excitation/inhibition balance in information processing and social dysfunction. *Nature* 477 171–178 10.1038/nature10360 21796121 PMC4155501

[B236] Yoshida M. Takayanagi Y. Inoue K. Kimura T. Young L. J. Onaka T. (2009). Evidence that oxytocin exerts anxiolytic effects via oxytocin receptor expressed in serotonergic neurons in mice. *J. Neurosci.* 29 2259–2271 10.1523/JNEUROSCI.5593-08.2009 19228979 PMC6666325

[B237] Zee P. C. Manthena P. (2007). The brain’s master circadian clock: implications and opportunities for therapy of sleep disorders. *Sleep Med. Rev.* 11 59–70 10.1016/j.smrv.2006.06.001 16973392

[B238] Zhang F. Gradinaru V. Adamantidis A. R. Durand R. Airan R. D. de Lecea L. (2010). Optogenetic interrogation of neural circuits: technology for probing mammalian brain structures. *Nat. Protoc.* 5 439–456 10.1038/nprot.2009.226 20203662 PMC4503465

[B239] Zhang J. Fan Y. Li Y. Zhu H. Wang L. Zhu M. Y. (2012). Chronic social defeat up-regulates expression of the serotonin transporter in rat dorsal raphe nucleus and projection regions in a glucocorticoid-dependent manner. *J. Neurochem.* 123 1054–1068 10.1111/jnc.12055 23061525 PMC3514584

[B240] Zheng H. Rinaman L. (2013). Yohimbine anxiogenesis in the elevated plus maze requires hindbrain noradrenergic neurons that target the anterior ventrolateral bed nucleus of the stria terminalis. *Eur. J. Neurosci.* 37 1340–1349 10.1111/ejn.12123 23368289 PMC3637934

[B241] Zorrilla E. P. Heilig M. de Wit H. Shaham Y. (2013). Behavioral, biological, and chemical perspectives on targeting CRF(1) receptor antagonists to treat alcoholism. *Drug Alcohol Depend.* 128 175–186 10.1016/j.drugalcdep.2012.12.017 23294766 PMC3596012

[B242] Zorrilla E. P. Koob G. F. (2010). Progress in corticotropin-releasing factor-1 antagonist development. *Drug Discov. Today* 15 371–383 10.1016/j.drudis.2010.02.011 20206287 PMC2864802

